# A Systems Biology Strategy Reveals Biological Pathways and Plasma Biomarker Candidates for Potentially Toxic Statin-Induced Changes in Muscle

**DOI:** 10.1371/journal.pone.0000097

**Published:** 2006-12-20

**Authors:** Reijo Laaksonen, Mikko Katajamaa, Hannu Päivä, Marko Sysi-Aho, Lilli Saarinen, Päivi Junni, Dieter Lütjohann, Joél Smet, Rudy Van Coster, Tuulikki Seppänen-Laakso, Terho Lehtimäki, Juhani Soini, Matej Orešič

**Affiliations:** 1 Research Unit, University Hospital of Tampere, Tampere, Finland; 2 Turku Centre for Biotechnology, University of Turku and Åbo Akademi University, Turku, Finland; 3 Department of Internal Medicine, University Hospital of Tampere, Tampere, Finland; 4 VTT Technical Research Centre of Finland, Espoo, Finland; 5 Department of Clinical Pharmacology, University of Bonn, Bonn, Germany; 6 Department of Pediatrics, Division of Pediatric Neurology and Metabolism, Ghent University Hospital, Ghent, Belgium; 7 Laboratory of Atherosclerosis Genetics, Department of Clinical Chemistry, Tampere, Finland; 8 Centre for Laboratory Medicine, University Hospital of Tampere, Tampere, Finland; 9 Medical School, University of Tampere, Tampere, Finland; Columbia University, United States of America

## Abstract

**Background:**

Aggressive lipid lowering with high doses of statins increases the risk of statin-induced myopathy. However, the cellular mechanisms leading to muscle damage are not known and sensitive biomarkers are needed to identify patients at risk of developing statin-induced serious side effects.

**Methodology:**

We performed bioinformatics analysis of whole genome expression profiling of muscle specimens and UPLC/MS based lipidomics analyses of plasma samples obtained in an earlier randomized trial from patients either on high dose simvastatin (80 mg), atorvastatin (40 mg), or placebo.

**Principal Findings:**

High dose simvastatin treatment resulted in 111 differentially expressed genes (1.5-fold change and *p*-value<0.05), while expression of only one and five genes was altered in the placebo and atorvastatin groups, respectively. The Gene Set Enrichment Analysis identified several affected pathways (23 gene lists with False Discovery Rate *q*-value<0.1) in muscle following high dose simvastatin, including eicosanoid synthesis and Phospholipase C pathways. Using lipidomic analysis we identified previously uncharacterized drug-specific changes in the plasma lipid profile despite similar statin-induced changes in plasma LDL-cholesterol. We also found that the plasma lipidomic changes following simvastatin treatment correlate with the muscle expression of the arachidonate 5-lipoxygenase-activating protein.

**Conclusions:**

High dose simvastatin affects multiple metabolic and signaling pathways in skeletal muscle, including the pro-inflammatory pathways. Thus, our results demonstrate that clinically used high statin dosages may lead to unexpected metabolic effects in non-hepatic tissues. The lipidomic profiles may serve as highly sensitive biomarkers of statin-induced metabolic alterations in muscle and may thus allow us to identify patients who should be treated with a lower dose to prevent a possible toxicity.

## Introduction

Large-scale clinical trials have shown that statins are effective and safe cholesterol lowering drugs [1]–[3]. Recently more patients have been titrated to higher doses of statins in order to reach the new goals of LDL-cholesterol lowering and achieve even greater reductions of atherosclerotic complications. However, aggressive treatment with high dosages increases the risk of statin-induced myopathy [4]. Elucidation of myopathy mechanisms and identification of patients likely not to tolerate the treatment is therefore of great clinical interest. In addition, comparison of different statin drugs used for aggressive treatment is essential. The currently used statins do have clear differences for instance in their pharmacokinetic properties [5], therefore it is likely that some of the statins at high dosages are more prone to have unexpected and unwanted effects in non-hepatic tissues.

We do know that some diseases such as hypothyroidism, liver dysfunction and diabetes increase the risk of muscle complications due to statin treatment [6]. Furthermore, exercise, alcohol, infections or underlying metabolic diseases seems to exacerbate this risk [7]. Under these circumstances development of myopathy may be exacerbated by interactions with statins [8]. Mukhtar and Reckless listed four potential myopathy mechanisms in their recent review: Depletion of intracellular cholesterol leading to calcium influx; inhibited protein synthesis, signal transduction and metabolism due to decreased mevalonate acid and its metabolite concentrations; reduced ubiquinone (coenzyme Q10) concentrations; and enhanced apoptosis [8]. Muscle biopsies obtained from patients with statin-induced myopathy without creatine kinase (CK) elevations have shown evidence of mitochondrial dysfunction, including abnormally increased lipid stores in muscles [9]. We observed decreased mitochondrial function in patients on high dose simvastatin treatment, with no signs of myopathy [10]. Later, we confirmed that high dose (80 mg) simvastatin affects muscle mitochondria by assessing a significant decrease in the muscle mitochondrial DNA (mtDNA) content during treatment (Schink et al, submitted). Thus, statins are causing unwanted mitochondrial effects and defective mitochondrial metabolism may already be involved during the early development of statin-induced myopathy when the currently used plasma CK measurements are not sensitive enough to identify these patients at risk of developing muscle damage.

Given the existing gap in knowledge and understanding of statin-induced muscle damage, we embarked in a systems biology approach aiming to gain insight into the mechanism and potential biomarkers of myopathy in the clinical setting. Of particular relevance is to gain new knowledge of signaling and metabolic pathways in muscle involved in myopathy and early molecular markers applicable in clinical setting. To address both objectives, gene expression profiling of muscle tissue is an obvious strategy of choice. As changes in plasma lipid composition are of particular interest in the study of statins, plasma lipidomics is one possible option to address the latter. Recent advances in liquid chromatography and mass spectrometry have empowered us with ability to reliably measure hundreds of lipid molecular species from biological samples in parallel [11], [12].

In this paper we report the study of muscle gene expression profiles in combination with plasma lipidome analysis before and during high dose statin treatment. The specimens were from patients who participated in our earlier controlled and randomized study comparing placebo and high dosages of atorvastatin and simvastatin [10]. The simvastatin treated patients were particularly unique and suitable for sensitive early marker discovery due to changes in their mitochondrial function, mtDNA and ubiquinone concentration in muscle. The systems biology approach allowed us to compare two widely used statins in terms of their effects on muscle gene expression and plasma lipidome. In addition, we were able to illuminate relevant biological pathways and to identify biomarker candidates related to unwanted and potentially toxic statin-induced changes in muscle metabolism.

## Methods

### Patients

Plasma samples from 37 subjects of an earlier study [10], focusing on the effect of high dose statin treatment on skeletal muscle metabolism, were used for plasma lipidome analysis; placebo (N = 11), simvastatin (N = 13), and atorvastatin (N = 14). The subjects aged between 45 and 69 years and their average serum total cholesterol concentration was 5.8±0.9 mmol/L and serum triglycerides below 4.5 mmol/L. Muscle specimens from eighteen age matched men being treated either with atorvastatin (n = 6), simvastatin (n = 6) or placebo (n = 6) were selected for genome wide expression analysis. Clinical parameters are available as Supporting Information Dataset S1 and Table S1.

The study patients had never been treated with statins before. They were instructed to adhere to their normal diet during the study. Patients with familial hypercholesterolemia and patients with serum total cholesterol>7.0 mmol/L in the initial screening were excluded. Other exclusion criteria were: use of concurrent lipid altering medication or antioxidant vitamins, renal or hepatic dysfunction, and use of medication known to affect metabolism of atorvastatin or simvastatin. The study protocol was accepted by the Ethics Committee of the Tampere University Hospital and written informed consents were obtained from all participants.

### Design

The original study was a randomized, double blind and placebo-controlled trial with three treatment groups: placebo, atorvastatin 40 mg/day, and simvastatin 80 mg/day. Placebo was simvastatin-matched, and to ensure also blinding of atorvastatin, all study drugs were supplied in sealed, identical, numbered containers. The duration of the follow-up was eight weeks. Muscle biopsies were obtained at baseline and at the end of the treatment period. Biopsies were taken from the lateral portion of the quadriceps femoris muscle in local anesthesia at about the mid-point between the greater trochanter and the knee joint with a biopsy needle (Tru-Cut, Baxter, McGaw Park, Ill., USA). The muscle specimens were frozen within 1–2 seconds in liquid nitrogen and stored at −80°C until analyzed. The blood sampling was performed in the Department of Clinical Chemistry, Tampere University Hospital by an experienced laboratory technician. Venous blood was drawn from the antecubital vein in sitting position after a twelve-hour fast and after 15 minute rest just before blood sampling. The blood was drawn into tubes containing EDTA, and plasma was separated after cooling by centrifugation at 2000 rpm for 10 minutes. The samples were stored at −70° until analyzed. Investigators performing the gene expression and lipidomics analyses were blinded until the analyses were done.

### Gene expression analyses

Microarray experiments were performed using Sentrix® Human-6 Expression BeadChips, analyzing over 46 000 known genes, gene candidates and splice variants (Illumina, San Diego, CA, USA) according to given instructions. The biopsy samples were homogenized using Ultra-Turrax (IKA Turrax T8/S8N-5G, IKA-Werke, Staufen, Germany). The total RNA was extracted using TRIzol (#15596-018, Invitrogen Corporation, Carlsbad, CA), DNase treatment and a second RNA purification by Qiagen kits (#74106, and, #79254, Qiagen GmbH, Hilden, Germany), all by given instructions.

A 200 ng aliquote of total RNA from each sample were amplified to cDNA using Ambion's Illumina RNA Amplification kit following the instructions (cat no I1755, Ambion, Inc., Austin, TX, USA). In vitro transcripiton (IVT) reaction of cDNA to cRNA was performed overnight (14h) including biotin-11-dUTP (PerkinElmer, cat no PC 3435-0402-Biotin-11-dUTP, >95%, NEL539001EA, PerkinElmer Life And Analytical Sciences, Inc., Boston, MA, USA) for labelling the cRNA product. Both before and after the amplifications the RNA/cRNA concentrations were checked with Nanodrop ND-1000 spectrophotometer (Nanodrop Technologies, Wilmington, DE, USA) and RNA/cRNA quality was controlled by BioRad's Experion Automated Electrophoresis System and RNA StdSens Analysis Kit (BioRad Laboratories, Inc., Hercules, CA, USA).

1500 ng of each sample cRNA was hybridized to Illumina's Sentrix® Human-6 Expression BeadChip arrays (Illumina, Inc., San Diego, CA, USA) at 55°C overnight (18 h) following the Illumina Whole-Genome Gene Expression Protocol for BeadStation (Doc. # 11176837 Rev. F, Illumina Inc.). Hybridized biotinylated cRNA was detected with 1 µg/ml Cyanine3-streptavidine (Amersham Biosciences #146065). BeadChips were scanned with Illumina BeadArray Reader.

Raw intensity data obtained from the Illumina™ platform were normalized with Inforsense KDE version 2.0.4 (Inforsense, London, UK) using quantile normalization method. The same software was also used for single-gene analyses including fold-change calculations and filtering the probes. The differences within the treatment group before and after the intervention were analyzed using the *t*-test statistic, with *p*-values calculated using 5000 permutations.

Pathway analysis of the expression data was performed using the Gene Set Enrichment Analysis (GSEA) implemented in javaGSEA application version 1.0 [13]. In order to avoid duplicates in the analysis, probes representing the same gene symbol in Illumina™ data were replaced with their average intensity before applying the GSEA. Gene sets for GSEA were taken from Database C2 of MSigDB version 1.0 of March 2005 [13]. Parameters used for the GSEA analysis are provided in Supporting Information Text S2. Gene expression data is available at Array Express web site (http://www.ebi.ac.uk/aerep/login; accession number E-TABM-116).

### RT-PCR analysis

The microarray expression results recorded in the simvastatin group (n = 5, for one case there was not enough muscle RNA for PCR) were verified by Real-Time Quantitative TaqMan PCR. Previously purified cRNA was used as starting material for cDNA synthesis. A 1000 ng–18 µl aliquote of cRNA was mixed with 1 µl Promega Random Primer (C1181, Promega U.S., Madison, WI, USA) and incubated in +70°C for 10 min. The following reagents were added leading to 25 µl total reaction volume: 1 µl of 10 µM dNTP blend (F09892, Applied Biosystems, Foster City, CA, USA), 1 µl of Promega M-MLV Reverse Transcriptase 200 U/µl (M3682) and 4 µl of M-MLV RT 5× reaction buffer. Finally the incubations were performed in the following order: 10 min in RT, 50 min in 45°C, and, 10 min in 70°C.

10 µl volume was used for PCR reaction, consisting of 2 µl aliquote of 1∶10 diluted cDNA sample, and, Abgene ABsolute 2× QPCR ROX mix (AB-1139, Abgene, Epsom, UK). The primer concentrations were 300 nM, probe concentrations for Universal Probe Library (Exiqon, Vedbæk, Denmark) probes 100 nM and for ordinary long probes 200 nM. Finally the PCR reactions were performed in rtPCR system (ABI Prism 7700 Sequence Detection System, Applied Biosystems) having the following PCR procedure: 95°C for 15 min, and 40 cycles of 95°C for 15 s and 60°C for 1 min. The primer and probe sequences are available upon request.

### Lipidomics analysis of plasma

An aliquot (10 µl) of an internal standard mixture containing 11 lipid classes, and 0.05 M sodium chloride (10 µl) was added to plasma samples (10 µl) and the lipids were extracted with chloroform/methanol (2∶1, 100 µl). After vortexing (2 min), standing (1 hour) and centrifugation (10000 RPM, 3 min) the lower layer was separated and a standard mixture containing 3 labeled standard lipids was added (10 µl) to the extracts. The sample order for LC/MS analysis was determined by randomization.

Lipid extracts were analysed on a Waters Q-Tof Premier mass spectrometer combined with an Acquity Ultra Performance LC™ (UPLC). The column, which was kept at 50°C, was an Acquity UPLC™ BEH C18 10×50 mm with 1.7 µm particles. The binary solvent system included A. water (1% 1 M NH_4_Ac, 0.1% HCOOH) and B. LC/MS grade (Rathburn) acetonitrile/isopropanol (5 2, 1% 1 M NH_4_Ac, 0.1% HCOOH). The gradient started from 65% A/35% B, reached 100% B in 6 min and remained there for the next 7 min. The total run time including a 5 min re-equilibration step was 18 min. The flow rate was 0.200 ml/min and the injected amount 0.75 µl. The temperature of the sample organizer was set at 10°C.

The lipid profiling was carried out on Waters Q-Tof Premier mass spectrometer using ESI+ mode. The data was collected at mass range of m/z 300–1200 with a scan duration of 0.2 sec. The source temperature was set at 120°C and nitrogen was used as desolvation gas (800 L/h) at 250°C. The voltages of the sampling cone and capillary were 39 V and 3.2 kV, respectively. Reserpine (50 µg/L) was used as the lock spray reference compound (5 µl/min; 10 sec scan frequency).

Data was processed using MZmine software version 0.60 [14]. Lipids were identified using internal spectral library. The normalization was performed using multiple internal standards as described in the Supporting Information Text S1. Only the identified lipid molecular species were included in further data analyses.

The Supporting Information Text S1, Figures S6–23 and Tables S8–11 also include general lipidomics platform characteristics such as internal and external standards used, calibration curves, dynamic ranges, recovery, variability, identification and quality control workflow, as well as illustrative spectra (MS and MS/MS) demonstrating how the specific species can be identified.

### Lipid nomenclature

Lipids from the lipidomic analysis were named according to Lipid Maps (http://www.lipidmaps.org) [15]. For example, lysophosphatidylcholine with 16∶0 fatty acid chain was named as monoacyl-glycerophosphocholine GPCho(16∶0/0∶0). In case the fatty acid composition was not determined, total number of carbons and double bonds was marked. For example, a phosphatidylcholine species GPCho(16∶0/20∶4) is represented as GPCho(36∶4). However, GPCho(36∶4) could also represent other molecular species, for example GPCho(20∶4/16∶0) or GPCho(18∶2/18∶2). Such mass isomers may be separated chromatographically, as shown in Supporting Information Figure S20A for two lysophosphatidylcholine species GPCho(17∶0/0∶0) and GPCho(0∶0/17∶0).

### Chemometric modeling and statistical analysis of lipidomics data

Partial least squares discriminant analysis (PLS/DA) [16], [17] was utilized as a supervised modeling method using SIMPLS algorithm to calculate the model [18]. As the total number of samples was insufficient for independent validation, no hold-out dataset was utilized for cross-validation. Instead, Venetian blinds cross-validation method [19] and *Q* ^2^ scores were used to optimize the model. Top loadings for latent variables associated with drug specific effects were reported. The VIP (variable importance in the projection) values [20] were calculated to identify the most important molecular species for the clustering of specific groups. Multivariate analyses were performed using Matlab version 7.2 (Mathworks, Inc.) and the PLS Toolbox version 3.5 Matlab package (Eigenvector Research, Inc.).

The regression of lipidomics data on muscle gene expression profiles was performed using the *lasso* method [21]. The *lasso* is a shrinkage regression method, similar to Ridge regression [22], which performs continuous variable selection causing some of the regression coefficients to be exactly zero. This reduces the variance of the regression estimates, which in the case of lipidomics data with large number of variables would otherwise be unacceptably high. Furthermore, the subset of lipids corresponding to non-zero coefficients can be considered as ‘the most important’ in explaining the muscle gene expression profiles. The *lasso* regression coefficients were calculated with the Least Angle Regression method [23] implemented in the R statistical language (package LARS). The corrected *R* ^2^ value and the Schwartz Criterion [24] were reported along with the measured and predicted gene expression values.

## Results

### Gene expression analysis reveals multiple upregulated pathways in the simvastatin group

In order to understand the pathways associated with statin response in muscle, we performed the whole genome microarray analysis of muscle biopsies. Microarray experiments were performed in 18 age-matched men (6 subjects from each group) who did not have any observed side effects such as muscle pain or CK elevations as a result of statin treatment. However, simvastatin treated men had substantial statin-induced unwanted and potentially toxic changes in muscle ubiquinone and mtDNA as reported earlier.

According to the used selection criteria for differentially expressed genes (1.5-fold change and *p*-value<0.05), expression of one gene was changed in the placebo group. Only modest changes were recorded in the atorvastatin group as expression of five genes was altered during the intervention. In the simvastatin group, however, expression of 111 genes changed (26 down-regulated and 85 up-regulated). Based on a hierarchical cluster analysis 20 genes were selected for further RT-PCR control. The following 5 genes were significantly upregulated: ALOX5AP (+3.6-fold, *p* = 0.041), CCL5 (+11.9-fold, *p* = 0.011), COL3A1 (+27.1-fold, *p* = 0.026), MYL5 (+8.0-fold, *p* = 0.021), MYBPH (+49.0-fold, *p* = 0.027).

As the recorded differences in single gene expressions were rather modest in general, we performed a Gene Set Enrichment Analysis [13] to identify globally affected metabolic pathways. The parameters of GSEA analysis are listed in Supporting Information Text S2. No pathways were affected significantly in the atorvastatin or placebo groups according to the criteria (False Discovery Rate *q*-value<0.25) recommended by Subramanian *et al.* [13]. However, in the simvastatin group 143 pathways were up-regulated (*q*<0.25) (Supporting Information Dataset S2). Due to the large number of affected pathways we limited our systematic analyses to the 23 most affected pathways (*q*<0.10) (Table 1).

**Table 1:**
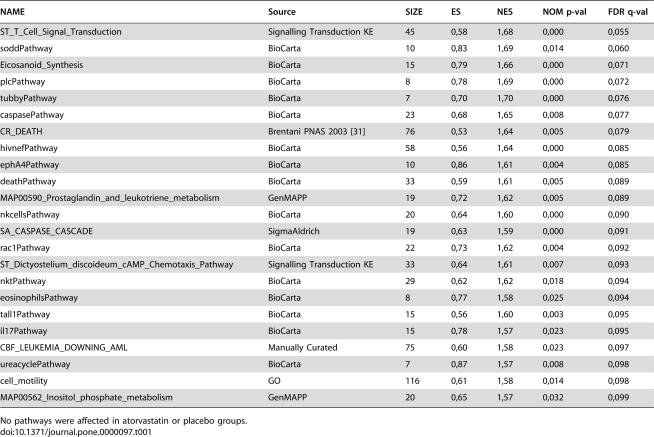
Affected pathways in simvastatin group as revealed by Gene Set Enrichment Analysis, with the False Discovery Rate (FDR) *q*-value cutoff of 0.1.

NAME	Source	SIZE	ES	NES	NOM p-val	FDR q-val
ST_T_Cell_Signal_Transduction	Signalling Transduction KE	45	0,58	1,68	0,000	0,055
soddPathway	BioCarta	10	0,83	1,69	0,014	0,060
Eicosanoid_Synthesis	BioCarta	15	0,79	1,66	0,000	0,071
plcPathway	BioCarta	8	0,78	1,69	0,000	0,072
tubbyPathway	BioCarta	7	0,70	1,70	0,000	0,076
caspasePathway	BioCarta	23	0,68	1,65	0,008	0,077
CR_DEATH	Brentani PNAS 2003 [31]	76	0,53	1,64	0,005	0,079
hivnefPathway	BioCarta	58	0,56	1,64	0,000	0,085
ephA4Pathway	BioCarta	10	0,86	1,61	0,004	0,085
deathPathway	BioCarta	33	0,59	1,61	0,005	0,089
MAP00590_Prostaglandin_and_leukotriene_metabolism	GenMAPP	19	0,72	1,62	0,005	0,089
nkcellsPathway	BioCarta	20	0,64	1,60	0,000	0,090
SA_CASPASE_CASCADE	SigmaAldrich	19	0,63	1,59	0,000	0,091
rac1Pathway	BioCarta	22	0,73	1,62	0,004	0,092
ST_Dictyostelium_discoideum_cAMP_Chemotaxis_Pathway	Signalling Transduction KE	33	0,64	1,61	0,007	0,093
nktPathway	BioCarta	29	0,62	1,62	0,018	0,094
eosinophilsPathway	BioCarta	8	0,77	1,58	0,025	0,094
tall1Pathway	BioCarta	15	0,56	1,60	0,003	0,095
il17Pathway	BioCarta	15	0,78	1,57	0,023	0,095
CBF_LEUKEMIA_DOWNING_AML	Manually Curated	75	0,60	1,58	0,023	0,097
ureacyclePathway	BioCarta	7	0,87	1,57	0,008	0,098
cell_motility	GO	116	0,61	1,58	0,014	0,098
MAP00562_Inositol_phosphate_metabolism	GenMAPP	20	0,65	1,57	0,032	0,099

No pathways were affected in atorvastatin or placebo groups.

### Serum lipidomics reveals drug-specific changes

**Figure 1:**
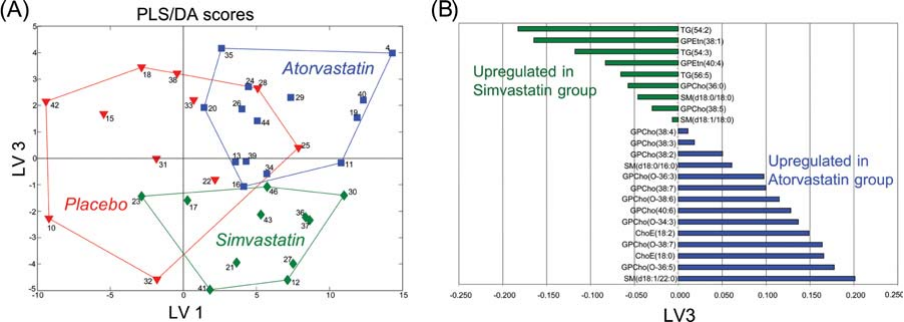
Partial least squares discriminant analysis (PLS/DA) of serum lipidomics data. Results after 8 week treatment from placebo (N = 11), atorvastatin (N = 14), and simvastatin (N = 12) groups, with 132 identified lipid species included in analysis as variables. For each molecular species and each subject, its level after the 8 week treatment period was scaled by subtracting its median level across all subjects prior to treatment and divided by corresponding standard deviation. Four latent variables were used in the model (*Q* ^2^ = 0.46). The labels are patient ID numbers. The lines outlining different groups are shown as a guide. (A) The scores for Latent Variables (LV) 1 and 3 reveal serum lipid changes specific to the statin treatment (LV1) as well as statin-specific changes (LV3). (B) Loadings on LV3 for most important lipids in simvastatin or atorvastatin groups selected by VIP analysis. Only lipids for which at least one of the two groups has VIP value greater than 2 are shown.

In order to investigate how the high dose statin treatment affects the plasma lipid profiles, we applied the UPLC/MS based lipidomics analysis, leading to a total of 132 identified lipid molecular species (data available as Supporting Information Dataset S3). Partial Least Squares Discriminant Analysis (PLS/DA) [17] revealed drug-specific changes in lipid profiles (Figure 1A). The PLS/DA model details are listed as Supporting Information Text S3 and Figure S1. The differences along the first latent variable (LV1), were associated with expected changes in triacylglycerols and cholesterol esters in agreement with the hypolipidemic effect expected from both drugs (Supporting Information Table S2). Specific differences between the simvastatin and atorvastatin lipid profiles were found in the third latent variable (LV3). Following VIP analysis, the most important lipid species were identified for each intervention group. The list of loadings in direction of atorvastatin-simvastatin differences (LV3) for most important lipids in simvastatin and atorvastatin groups is shown in Figure 1B. Notably, the main plasma lipid profile differences between the two statins can be considered as lipid-class specific, with specific upregulation of several phosphatidylethanolamines species and selective pools of long chain triacylglycerols. Similarly, downregulation of ether phosphocholines and cholesterol esters were observed in the simvastatin group compared to the atorvastatin group.

### Combined lipidomics and gene expression

We wanted to identify if any of the lipidomic changes in plasma could be used as a marker of altered gene expression in muscle in the high dose simvastatin intervention group. Therefore we investigated if any of these gene expression changes were associated with the differences observed in the serum lipidome.

**Figure 2:**
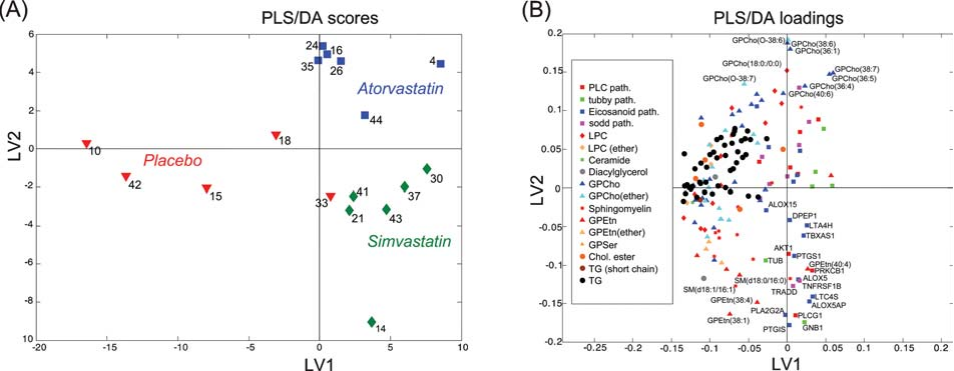
PLS/DA analysis on combined muscle gene expression and serum lipid data. Results after intervention for the subjects from placebo (N = 5), atorvastatin (N = 6), and simvastatin (N = 6) groups. Total 38 genes from four enriched pathways and 132 lipids were included in the analysis as variables. Data was autoscaled prior to multivariate analysis. Three latent variables were used in the model (*Q* ^2^ = 0.50). The labels are patient ID numbers. (A) The PLS/DA score plot reveals treatment-specific differences between the treatments are observed in molecular profiles after intervention. (B) Loadings for the first two latent variables reveal plasma lipid classes and muscle pathways associated with specific interventions. LPC is shorthand for lysophosphatidylcholine (for example GPCho(18∶0/0∶0)).

We selected a subset of genes based on GSEA analysis. Specifically genes from PLC, tubby, eicosanoid biosynthesis, and sodd pathways were chosen, based on their ranking as 2nd to 5th on FDR *q*-value. The top scored pathway “ST_T_Cell_Signal_Transduction” was not selected for further analysis since it is less pathway-specific than the other four and overlaps with the PLC pathway. The PLS/DA analysis on combined muscle gene expression (38 transcripts) and plasma lipid profile data revealed clear differences between the three treatment groups (Figure 2A). The PLS/DA model details are listed as Supporting Information Text S4. Simvastatin treatment was primarily associated with the gene expression changes in multiple genes involved in eicosanoid synthesis pathways as well as changes in multiple phosphatidylethanolamine and sphingomyelin molecular species (Figure 2B). Since the PLS analysis maximizes the product of variance matrix of measured variables (e.g. combined gene expression and lipid profile data) and correlation of measured data with properties of interest (e.g. treatment groups), our results indicate that there is a high degree of correlation between the upregulated genes (pathways) in skeletal muscle and specific lipids plasma changes in the simvastatin group.

**Figure 3:**
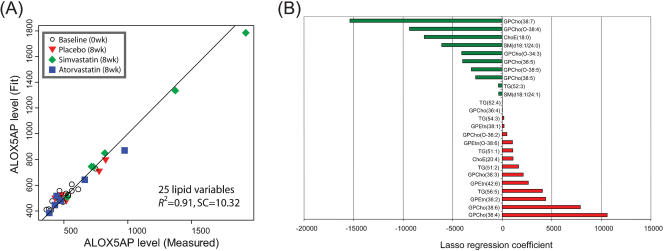
Regression of plasma lipid data on arachidonate 5-lipoxygenase activating protein (ALOX5AP) muscle gene expression profile using the *lasso* method. 25 lipid variables were chosen to build the regression model. (A) ALOX5AP expression values as predicted by the model. (B) Regression coefficients for the lipid species selected by *lasso*.

The results from combined gene expression and lipid PLS/DA analysis raise the possibility that plasma lipid biomarkers may be found for the pathway changes observed in the muscle. In order to investigate this possibility, we performed regression analysis of plasma lipid profile data on a muscle selected marker gene expression profile. We chose arachidonate 5-lipoxygenase activating protein (ALOX5AP, Uniprot ID: P20292) as the marker for simvastatin dysregulated pathway changes in muscle. The ALOX5AP gene was selected based on high VIP scores in multiple PLS/DA analyses, PCR validated significant fold change in the simvastatin group, and its well known (pro-inflammatory) biological role [25]. As the main goal of this analysis was discovery of potential plasma molecular markers for statin induced muscle toxicity, we applied a shrinkage regression method *lasso* [21] aiming to find a subset of plasma lipids predictive of specific gene expression levels in skeletal muscle. Figure 3A shows the results of the *lasso* model for *NZ* = 25 non-zero lipid variables. The variables and their coefficients are shown in Figure 3B. The *lasso* analysis was also performed for *NZ* = 5, 10, 15, and 20 non-zero variables (Supporting Information Tables S3–S7 and Figures S2–S5). Our analyses identified ALOX5AP gene expression in muscle had a high positive regression coefficients with plasma levels of phosphatidylethanolamine (42∶6) and negative for the cholesterol ester ChoE(18∶0), i.e. both type of lipids were selected as the non-zero variables in all regression analyses, with consistent results. Also in all analyses except *NZ* = 5, the sphingomyelins SM(d18∶1/24∶0) and SM(d18∶1/24∶1) showed negative regression coefficients (Figure 2B). Also the ether phosphocholines were selected with consistently negative coefficients, in agreement with PLS/DA analyses (Figures 1B and 2B).

## Discussion

Our systems biology strategy using combined gene expression and lipidomics analyses revealed that simvastatin at high doses induces significant changes in the expression of multiple genes controlling metabolic and inflammatory pathways in a non-hepatic tissue. This observation markedly contrast with the minimum gene expression changes observed in skeletal muscle with atorvastin 40mg. Our studies also revealed novel plasma biomarker candidates for safety assessment of statin treatment before recorded changes in muscle metabolism become clinically evident.

Similar to an earlier report [26], expression of genes related to cholesterol metabolism or mevalonate pathway were only modestly affected by statins in the present study. Thus, our data do not directly support the view that statins would cause mitochondrial dysfunction by reducing ubiquinone, a mitochondrial coenzyme with a cholesterol synthetic pathway derived side-chain, due to inhibition of HMG-CoA reductase in the muscle. Similarly we were not able to provide evidence that statins would lead to inhibition of protein synthesis, signal transduction and metabolism due to decreased muscle mevalonate acid. Since our patients did not have any signs of clinical myopathy and muscle damage, we were not able to judge the significance of early proapoptotic markers during the course of the myopathy. However, in the GSEA analysis several pro-apoptosis pathways already appeared with significant FDR *q*-values at these early stages and, therefore, the present results support the role of pro-apoptosis pathways in statin myotoxicity. Furthermore, the hypothesis of an increased Ca2+ influx as a mediator of statin induced toxicity is supported by the significant up-regulation of phospholipase C pathway and by the dysregulation of genes encoding for calcium binding proteins (Supporting Information Dataset S2) in the present study. Another hallmark of high dose simvastatin effect in muscle is the activation of pro-inflammatory pathways such as eicosanoid synthesis. However, the present results cannot reveal the actual trigger leading to impaired mitochondrial function and induction of these proinflamatory pathways.

Atorvastatin and simvastatin treatments also resulted in specific plasma lipidome profiles. Thus, lipidomics analysis may have the potential of providing individualized specific lipid lowering agents suitable to individual patients after the significance of these novel lipid biomarkers is elucidated. The role of sphingolipids as independent predictors of coronary artery disease has been previously suggested [27]. However, our results demonstrate that plasma sphingomyelin changes in response to statin therapy varies for different sphingomyelin molecular species. This raises important questions about the biological significance of these molecular species.

We also identified ether phospholipids as another type of lipids differentially regulated by simvastatin. Interestingly plasmalogens, a most abundant sub-class of ether phospholipids, have been associated with protection against oxidative damage [28], [29]. Their observed decrease (and negative correlation with the ALOX5AP expression) following simvastatin treatment may thus be functionally linked to increased oxidative stress and inflammation in muscle. Phosphatidylethanolamines were specifically dysregulated by simvastatin. The significance of this observation remains to be elucidated but it is interesting that a link between the Leukotriene B4 (a lipid synthesised via ALOX5AP) and the release of arachidonate from phosphatidylethanolamine in human neutrophils has been established [30]. All together these data indicate that in parallel with specific gene expression changes in skeletal muscle, treatment with simvastatin was also associated with parallel changes in plasma of lipidic proinflamatory markers.

One limitation of the present study is the relatively small sample size due to obvious limitations in the number of muscle specimens obtained from patients. However, the conclusions of the results are strengthened by the combined genomic and lipidomic analyses. Although it may considered a potential weakness of the study, we decided not include at this stage any patients with acute proven myopathy. The rational for this is that analysis of gene expression profiles in specimens obtained from patients during acute muscle events (unpublished results) revealed hundreds of different genes affected in the context of muscle damage, making it rather difficult to analyze the results as directly related to the statin treatment or establish their potential use as early markers of myopathy. Therefore our strategy of investigating individuals with well-defined statin-induced mitochondrial defects during a randomized trial allowed us to identify markers with potential diagnostic and prognostic value. In conclusion, the combined analyses of gene expression and lipidomics profiles in asymptomatic statin treated individuals revealed that: a) simvastatin 80 mg induces specific gene expression and lipid changes compared to equally efficient atorvastatin treatments and b) that our combined transcriptomic lipidomic analysis provides *bona fide* sensitive biomarkers of statin induced metabolic changes in muscle potentially useful to identify patients at risk early enough to prevent actual muscle damage. These biomarkers are now available for further validation in patients with proven statin-induced myopathy.

## Supporting Information

Text S1 Lipidomics platform characteristics and quality control.
(0.05 MB DOC)

Text S2 Parameters for the GSEA analysis.
(0.04 MB DOC)

Text S3 Plasma lipidomics PLS/DA model details, corresponding to the Figure 1 of the paper.
(0.03 MB DOC)

Text S4 Combined plasma lipidomics and muscle gene expression PLS/DA model details, corresponding to the Figure 2 of the paper.
(0.03 MB DOC)

Table S1 Characteristics of the patients selected for tissue gene expression analysis at baseline and at the end of the study.
(0.03 MB DOC)

Table S2 The top loads from the lipidomics analysis, ranked by increasing first latent variable (differentiating between the placebo and statin-treated groups as shown in Figure 1). LV1 therefore describes the lipid changes common to both statins. Negative LV1 values correspond to upregulation in placebo group. As expected the most abundant triacylglycerol, cholesterol ester, and phospholipids species are downregulated following the statin treatment.
(0.05 MB DOC)

Table S3 Lasso regression of plasma lipids on muscle ALOX5AP expression for NZ = 5 lipid variables. Lipid identifiers and their regression coefficients are listed.
(0.04 MB DOC)

Table S4 Lasso regression of plasma lipids on muscle ALOX5AP expression for NZ = 10 lipid variables. Lipid identifiers and their regression coefficients are listed.
(0.04 MB DOC)

Table S5 Lasso regression of plasma lipids on muscle ALOX5AP expression for NZ = 15 lipid variables. Lipid identifiers and their regression coefficients are listed.
(0.04 MB DOC)

Table S6 Lasso regression of plasma lipids on muscle ALOX5AP expression for NZ = 20 lipid variables. Lipid identifiers and their regression coefficients are listed.
(0.04 MB DOC)

Table S7 Characteristics of the lasso regression (plasma lipids on muscle ALOX5AP expression) models for different number of non-zero regression coefficients.
(0.04 MB DOC)

Table S8 Standard compounds used in lipidomics platform, their monoisotopic masses, the fragments used and average retention times.
(0.06 MB DOC)

Table S9 Recovery data for three internal lipid standards.
(0.05 MB DOC)

Table S10 Repeatability of the UPLC/MS runs determined from 10 successive injections, as determined from the same liver extract.
(0.05 MB DOC)

Table S11 Repeatability of the analysis including standard addition, extraction and UPLC/MS analysis.
(0.05 MB DOC)

Figure S1 PLS/DA model for lipidomics analysis (corresponding to Figure 1). Root-Mean-Square Error of Cross-Validation (RMSECV) for three intervention groups. Four latent variables were chosen for the model.
(0.07 MB TIF)

Figure S2 Lasso regression of plasma lipids on muscle ALOX5AP expression for NZ = 5 lipid variables. ALOX5AP expression values as predicted by the model.
(0.05 MB TIF)

Figure S3 Lasso regression of plasma lipids on muscle ALOX5AP expression for NZ = 10 lipid variables. ALOX5AP expression values as predicted by the model.
(0.05 MB TIF)

Figure S4 Lasso regression of plasma lipids on muscle ALOX5AP expression for NZ = 15 lipid variables. ALOX5AP expression values as predicted by the model.
(0.05 MB TIF)

Figure S5 Lasso regression of plasma lipids on muscle ALOX5AP expression for NZ = 20 lipid variables. ALOX5AP expression values as predicted by the model.
(0.05 MB TIF)

Figure S6 Total ion UPLC/MS chromatograms for different extractions of a control serum sample, with chloroform:methanol in ratios 1:9 - 2:1.
(0.07 MB TIF)

Figure S7 Setup of lipidomics runs.
(0.11 MB TIF)

Figure S8 Variability of retention time for lysophosphatidylcholine GPCho(16:0/0:0) over a period of 18 months as determined from different experiments across different tissues and analytical UPLC C18 columns.
(0.07 MB TIF)

Figure S9 An example of typical UPLC/MS total ion chromatograms (TIC) from a serum lipid extract in ESI+ and ESI- mode.
(0.06 MB TIF)

Figure S10 Two dimensional view of typical lipidomics spectra.
(0.28 MB TIF)

Figure S11 Quality control flow-chart for metabolomics data processing.
(0.12 MB TIF)

Figure S12 Lipidomics data processing and identification workflow.
(0.31 MB TIF)

Figure S13 Calibration curves for internal lipid standards (added to samples prior to extraction) as determined from human serum total lipid extracts. (A) Concentration range 0.5-2.5 *μ*g/ml. (B) Concentration range10-250 *μ*g/ml. Lines drawn as guides.
(0.09 MB TIF)

Figure S14 Calibration curves for labeled external lipid standards (added to samples after the extraction) as determined from human serum total lipid extracts. (A) Concentration range 0.5-2.5 *μ*g/ml. (B) Concentration range10-250 *μ*g/ml.
(0.13 MB TIF)

Figure S15 MS/MS spectra of phosphatidylethanolamine GPEtn(17:0/17:0) in ESI+ and ESI-.
(0.07 MB TIF)

Figure S16 (A) Mass spectra of selected cholesteryl esters in human serum. (B) The extracted ion chromatogram of cholesteryl esters (as detected at m/z 369) in serum LDL-fraction and in total serum.
(0.11 MB TIF)

Figure S17 ESI+ MS and MS/MS spectra of ceramide Cer(d18:1/17:0).
(0.09 MB TIF)

Figure S18 ESI+ MS and MS/MS spectrum of diacylglycerol DG(17:0/17:0).
(0.06 MB TIF)

Figure S19 ESI+ MS/MS spectrum of triacylglycerol TG(17:0/17:0/17:0), with base peak m/z 903 and the m/z 603 peak corresponding to neutral loss of fatty acyl 17:0.
(0.05 MB TIF)

Figure S20 (A) Mass spectra of lysophosphatidylcholine GPCho(17:0/0:0), two separate chromatographic peaks in serum lipid extracts (sn-2 and sn-1). (B) ESI+ MS/MS spectrum of GPCho(16:0/0:0), with the characteristic m/z 184 peak.
(0.11 MB TIF)

Figure S21 (A) ESI+ MS and MSMS spectra of GPCho(16:0/18:0). (B) ESI- MS spectrum of GPCho(16:0/18:0).
(0.10 MB TIF)

Figure S22 ESI+ MS/MS spectrum of the ethanolamine plasmalogen GPEtn(O-18:1(1Z)/20:4), with the characteristic m/z 361 and m/z 392.
(0.07 MB TIF)

Figure S23 (A) A mixture of ESI+ MS spectra from a sphingomyelin extract. (B) ESI+ MS/MS spectra of two sphingomyelin species.
(0.11 MB TIF)

Dataset S1 Clinical background data for the subjects included in the study.
(0.02 MB XLS)

Dataset S2 Results of GSEA analysis.
(0.26 MB XLS)

Dataset S3 Lipidomics data.
(0.20 MB XLS)
