# Supplementary material for: A Systems Biology Strategy Reveals Biological Pathways and Plasma Biomarker Candidates for Potentially Toxic Statin-Induced Changes in Muscle
Source: PLoS One. 2006 Dec 20;1(1):e97. doi: 10.1371/journal.pone.0000097 (PMC1762369; doi:10.1371/journal.pone.0000097)
Supplement: Text S1 — Lipidomics platform characteristics and quality control. (0.05 MB DOC) [file pone.0000097.s001.doc]

**Storage of samples.** All serum samples are stored in a freezer at -80°C before sample preparation steps. The serum samples are let to melt at room temperature.

**Internal standards for serum.** A standard mixture containing equal amounts (0.1 mg/ml chloroform:methanol 2:1) of 11 lipid classes with heptadecanoic acid (17:0) as the acyl chain is prepared from separate stock solutions (1 mg/ml). The mixture is further diluted to 1:10 giving the final concentrations of 10 µg/ml. For serum or plasma samples, 10 µl aliquots are used. The standard mixture contains GPCho(17:0/0:0), GPCho(17:0/17:0), GPEtn(17:0/17:0), GPGro(17:0/17:0)[rac], Cer(d18:1/17:0), GPSer(17:0/17:0), GPA(17:0/17:0) and D-*erythro*-Sphingosine-1-Phosphate (C17 Base) from Avanti Polar Lipids and MG(17:0/0:0/0:0)[rac], DG(17:0/17:0/0:0)[rac] and TG(17:0/17:0/17:0) from Larodan Fine Chemicals.

**Lipid extraction.** Serum samples (10 µl) are diluted with 10 µl of 0.15 M (0.9%) sodium cloride and extracted with a mixture of chloroform and methanol (2:1; 100 µl, HPLC-grade, Rathburn).

As part of method development, different ratios of chloroform and methanol (1:9, 1:5, 1:2, 1:1, 2:1) were tested with a control serum sample, the total ion chromatographic profiles of the experiment are shown in Supplementary Figure S6. The extreme ratios of 1:9 and 1:5 are difficult in respect to the separation of the phases as they are mainly extracting phospholipids (Retention time ~ 5.5-7.0 min). The amount of sodium chloride solution was adjusted to obtain proper separation of the polar and non-polar phases.

Serum samples are homogenized by vortexing (5 s / sample). The extraction time is 0.5 h in case of serum and 1 h for other tissues. The samples are centrifuged at 10 000 RPM for 3 min by an Eppendorf centrifuge, and an exact aliquot of the lower layer (60 µl for serum) is transferred into an HPLC vial insert (150 µl).

**External standards.** An external standard mixture containing 3 labelled standards is added to the lipid extracts (10 µl) before analysis in order to control the extraction process. The standard solution contains 10 µg/ml (in chloroform:methanol 2:1) GPCho(16:0/0:0-D3), GPCho(16:0/16:0-D6) and TG(16:0/16:0/16:0-^13^C3), all from Larodan Fine Chemicals.

**Storage of lipid extracts before and during LC-MS runs.** Samples are stored in inserts and vials under gas tight caps in a freezer at -20°C. During runs the sample organizer was kept at +10°C since at lower temperatures some precipitation of lipids occurred. Evaporation of the solvent in the sample organizer was smaller when using isopropanol as the final solvent.

**Setting up lipidomics runs.** The total workflow of lipid analysis is shown in Supplementary Figure S7. The sample analysis order was randomized, and the control serum sample and standard mixture runs were regularly analyzed for quality control.

**Chromatography.** Lipid extracts are analysed on a Waters Q-Tof Premier mass spectrometer combined with an Acquity Ultra Performance LC™ (UPLC). The column, which is kept at 50°C, is an Acquity UPLC^TM^ BEH C18 10 × 50 mm with 1.7 µm particles. The binary solvent system includes A. water (1% 1M NH_4_Ac, 0.1% HCOOH) and B. acetonitrile/ isopropanol (5:2, 1% 1M NH_4_Ac, 0.1% HCOOH). The linear gradient starts from 65% A/ 35% B, reaches 100% B in 6 min and remains at this level for the next 7 min. The total run time including a 5 min re-equilibration step is 18 min. The flow rate is 0.200 ml/min and the injected amount 0.75 µl. The temperature of the sample organizer is set to 10°C.

The solvents (acetonitrile, isopropanol) are LC/MS grade (Rathburn), likewise NH_4_Ac (Merck) and HCOOH (Fluka). The water used in sample preparation and in UPLC solvents is ultra-pure grade. A solvent run is performed always after 5 lipid runs and the column is purified e.g. by flowing solvent B through the column overnight at a flow rate of 0.05 ml/min. In the UPLC system, 10% acetonitrile is used as the weak wash solvent and 70% acetonitrile as the strong wash solvent for the needle, and 1% acetonitrile is used as the seal wash solvent. The pressure in the column changes from about 8000 psi (max pressure allowed 15 000 psi) to 3600 psi during the run. By using elevated column temperature (50°C) and by keeping the proportion of isopropanol at 28% in solvent B the pressure cycle remains very repeatable also when changing the column after 400-600 runs. The variation in the retention time of GPCho(16:0/0:0), for example, is 1.25% as tested for multiple tissue or cell types over an extended period of time (18 months) for multiple columns (Supplementary Figure S8).

**Mass spectrometry.** The profiling of lipid extracts is carried out on Waters Q-Tof Premier mass spectrometer using electrospray ionization in positive ion mode (ESI+). The data is collected in centroid mode using extended dynamic range at mass range of m/z 300-1200 with a scan duration of 0.2 s. The interscan time is 0.02 s. The sample amounts are optimized to keep the order of magnitude of the abundances (TIC) at a linear range (<10^4^).

The source temperature is set at 120°C and nitrogen is used as the desolvation gas (800 L/h) at 250°C. The voltage of the sampling cone is 39 V and that of the capillary 3.2 - 3.5 kV to obtain maximum sensitivity. Reserpine at concentration of 50 ng/ml is used as the lock spray reference compound (m/z 609.2812) at a flow rate of 10 µl/min (Harvard PHD 2000 Pump) and the measurement is done at 10 s scan frequency. Calibration of the instrument is performed by using sodium formate.

In negative ion mode (ESI-) the voltages of the capillary and sampling cone are 2.7 kV and 72 V, respectively. The temperatures, gas flows and scan times are the same as in ESI+ mode. The lock spray mass for reserpine in ESI- is 607.2656.

A typical total ion chromatogram from human serum lipid extract in ESI+ and ESI- mode is presented in Supplementary Figure S9 and a typical two-dimensional representation of the spectra in Supplementary Figure S10.

**Data processing and quality control.** The raw data files obtained from LC/MS runs are converted to vendor-independent NetCDF (network Common Data Format) format. MZmine software version 0.60 (http://mzmine.sourceforge.net/) is applied for data processing. MZmine processing consists of four phases: detection, alignment, filtering and gap filling, each of which is controlled by several parameters. In the detection phase peaks in each sample are marked. Then the marked peaks with similar values of m/z and rt are aligned across several samples, and those peaks that appear only in few samples are filtered out. Finally, gaps corresponding to peaks that are missing from some samples but are present in the other samples are filled in. The result is output as a single alignment list showing peaks with different m/z-rt pairs on rows and the peak heights in different samples on the columns.

Quality of the results is monitored by checking that standard peaks are found (Supplementary Figure S11). Since standards are added into each sample they should not, in principle, be missing in any of the samples. In practise, however, standard peaks are frequently missing from many samples in the alignment list, e.g., due to unsuccessful alignment of peaks with deviating positions, and their values are thus imputed by the gap filling method. The aim of the quality control is to find such set of parameters for the MZmine software that as many standards as possible are found and aligned correctly, without using the gap filling, in as many samples in the alignment list as possible. Search for such parameters is done by trial and error, iteratively tuning the parameters until an acceptable alignment list results. In the end, peaks in the accepted alignment list are cleaned from isotopes with a Matlab code (available upon request), and the remaining base peaks are identified by their m/z and rt values using a database that has been built in-house. This results in the final list containing the working data.

**Identification of lipid subspecies.** Tandem mass spectrometry in both positive and negative ion modes is used for identification. The collision energy is ramped from 10 to 35 V and the mass range starts from m/z 100. The ESI- mode is primarily directed to identification of fatty acyl chains in phospholipids. The other conditions are as in the basic methods described above.

A lipid compound library using the LIPID MAPS nomenclature has been developed and applied for the automatic identification of lipids from lipidomics experiments. In addition to the masses, the database is utilizing isotopic distribution for every molecular species, and also the retention times obtained with the present UPLC-MS platform. Total informatics workflow is shown in Supplementary Figure S12.

**Quantification.** The added standards, used for identification and quantification, are picked from processed data according to their masses and expected retention times (Supplementary Table S8). The normalization of lipidomics data are performed as follows: all monoacyl lipids except cholesterol esters, such as monoacylglycerols and monoacyl-glycerophospholipids, were normalized with GPCho(17:0/0:0) internal standard, all diacyl lipids except ethanolamine phospholipids were normalized with GPCho(17:0/17:0), the diacyl ethanolamine phospholipids were normalized with GPEth(17:0/17:0), and the triacylglycerols and cholesterol esters with TG(17:0/17:0/17:0). Other molecular species were normalized by GPCho(17:0/0:0) for retention time <310seconds, GPCho(17:0/17:0) for retention time between 310s and 450s, and TG(17:0/17:0/17:0) for higher retention times. The concentration unit shown in figures is in μmol/l, unless shown otherwise.

**Linearity.** Calibration curves were determined for standard compounds used in serum analyses. For the serum internal standard mixture (Supplementary Figures S13A-B) as well as for the labeled standards (Supplementary Figs. S14A-B), the linearity was determined from serum at concentration range 0.5 - 250 µg/ml using 6 different levels (0.5, 1, 2.5, 10, 50 and 250 µg/ml). At each concentration, 3 replicate samples were analyzed and the mean value is reported.

**Extraction recovery.** Extraction recoveries were determined at 5 different (see above) concentration levels for GPCho(17:0/0:0), GPCho(17:0/17:0) and TG(17:0/17:0/17:0) added to liver tissue samples. The peak heights were compared to corresponding labelled standards (Supplementary Table S9) which were added to the samples after extraction. At all concentrations, 3 replicate samples were extracted and analyzed. The amount of the external standard mixture was unchanged (concentration level 1µg/ml) in all samples. At high concentration, recovery for triacylglycerol TG(17:0/17:0/17:0) is notably lower than for the phospholipids.

**Repeatability.** The repeatability of the UPLC/MS analysis was calculated from 10 successive runs, e.g. by multiple injections of the same liver tissue sample. In order for the repeatability to reflect the variation both in analytical and data processing stages, the absolute peak heights of the standards were used as determined by automated MZmine software. The ratios between the lipid internal standards and the labeled standards were calculated (Supplementary Table S10). For the 8 internal standards the coefficients of variation (CV %) were 4.0 - 8.6.

The repeatability of the sample preparation (standard addition, extraction, labeled standard addition) and analysis (UPLC/MS run, MZmine data processing) was determined by preparing 6 samples from liver tissue (Supplementary Table S11). The CV % values ranged between 3.3 - 12.2.

**Detection limit**. The detection limit in routine runs for the S/N ratio of 3 was 0.2 ng for GPCho(17:0/0:0), 1.0 ng for Cer(d18:1/17:0), 0.12 ng for GPEtn(17:0/17:0), 0.02 ng for GPCho(17:0/17:0) and 0.3 ng for TG(17:0/17:0/17:0).

**Illustrative MS- and MS/MS-spectra of lipid compounds in ESI+ and ESI- mode.** An example of ESI+ and ESI- spectrum is given in Supplementary Figure S15. The compound is GPEtn(17:0/17:0). In positive ion mode the M+1 peak is the base peak and additionally the fragment (m/z 579) resulting from the neutral loss of m/z 141 is very strong. In negative ion mode, the M-1 peak is seen, and the base peak in this case is the m/z 269 showing that heptadecanoic acid (C17:0) is the only esterified fatty acid in this compound.

The mass spectra of cholesteryl esters and their profile in human serum and in serum LDL-fraction are shown in Supplementary Figure S16.

Supplementary Figure S17 is shows the ESI+ MS and MS/MS spectra of ceramide lipid species Cer(17:0/17:0), with the characteristic m/z 264 peak.

Supplementary Figure S18 shows the MS and MS/MS (ESI+) spectra of diacylglycerol DG(17:0/17:0)[rac]. In addition to the M+1 peak (m/z 597) the major fragments are m/z 614 (M+18) and m/z 579 (M-17).

Supplementary Figure S19 contain the ESI+ MS and MS/MS spectra of triacylglycerol TG(18:1/18:1/18:1). The m/z 603 in MS/MS spectrum is due to the neutral loss of 18:1 fatty acid.

The ESI+ MS of *sn*-2 and *sn*-1 isomers of lysophosphatidylcholine GPCho(17:0/0:0) is shown in Supplementary Figure S20A. Notably, the retention times between the molecular species differ (beyond the measured within-species retention time variation), with the LPC in *sn*-2 having shorter retention time. The MS/MS analysis of GPCho(16:0/0:0) from Supplementary Figure S20B gives the characteristic m/z 184 peak.

An example of phosophatidylcholine GCCho(16:0/18:0) with different acyl groups is seen in Supplementary Figs. S21A-B. The ESI+ MS and MS/MS spectra show the cleavage of choline group (m/z 184), and ESI- spectrum gives the data of the fatty acyl chains (m/z 255 for the 16:0 and m/z 283 for the 18:0).

Supplementary Figure S22 shows ESI+ MS/MS spectra of ethanolamine plasmalogen GPEtn(O-18:1(1Z)/20:4) in human serum. Supplementary Figure S23 shows examples of ESI+ MS spectra from sphingomyelin mixture and MS/MS spectra for two SM species
