# Supplementary material for: A Systems Biology Strategy Reveals Biological Pathways and Plasma Biomarker Candidates for Potentially Toxic Statin-Induced Changes in Muscle
Source: PLoS One. 2006 Dec 20;1(1):e97. doi: 10.1371/journal.pone.0000097 (PMC1762369; doi:10.1371/journal.pone.0000097)
Supplement: Text S2 — Parameters for the GSEA analysis. (0.04 MB DOC) [file pone.0000097.s002.doc]

**Placebo group**

producer_class xtools.gsea.Gsea

producer_timestamp 1149104495046

param cls

o_Before

param plot_top_x 20

param norm meandiv

param save_rnd_lists false

param median false

param scoring_scheme weighted

param make_sets true

param gui true

param metric Signal2Noise

param rpt_label Placebo

param help false

param order descending

param permute phenotype

param rnd_type no_balance

param set_min 0

param sort real

param nperm 5000

param rnd_seed 149

param zip_report false

param set_max 500

**Simvastatin group**

producer_class xtools.gsea.Gsea

producer_timestamp 1148977892535

param cls

param plot_top_x 20

param norm meandiv

param save_rnd_lists false

param median false

param scoring_scheme weighted

param make_sets true

param gui true

param metric Signal2Noise

param rpt_label Sim

param help false

param order descending

param permute phenotype

param rnd_type no_balance

param set_min 0

param sort real

param nperm 5000

param rnd_seed 149

param zip_report false

param set_max 500

**Atorvastatin group**

producer_class xtools.gsea.Gsea

producer_timestamp 1149053189524

param cls

param plot_top_x 20

param norm meandiv

param save_rnd_lists false

param median false

param scoring_scheme weighted

param make_sets true

param gui true

param metric Signal2Noise

param rpt_label Ator

param help false

param order descending

param permute phenotype

param rnd_type no_balance

param set_min 0

param sort real

param nperm 5000

param rnd_seed 149

param zip_report false

param set_max 500
