# Supplementary material for: A Systems Biology Strategy Reveals Biological Pathways and Plasma Biomarker Candidates for Potentially Toxic Statin-Induced Changes in Muscle
Source: PLoS One. 2006 Dec 20;1(1):e97. doi: 10.1371/journal.pone.0000097 (PMC1762369; doi:10.1371/journal.pone.0000097)
Supplement: Text S3 — Plasma lipidomics PLS/DA model details, corresponding to the Figure 1 of the paper. (0.03 MB DOC) [file pone.0000097.s003.doc]

The model parameters retrieved from the PLS Toolbox:

X-block: Xd1 36 by 132

Included: [ 1-12 14-37 ] [ 1-132 ]

Preprocessing: None

Y-block: y 36 by 3

Included: [ 1-12 14-37 ] [ 1-3 ]

Preprocessing: Autoscale

Num. LVs: 4

Cross validation: venetian blinds w/ 6 splits

RMSEC: 0.10545 0.02 0.068182

RMSECV: 0.39273 0.34727 0.40909

Percent Variance Captured by Regression Model

-----X-Block----- -----Y-Block-----

Comp This Total This Total

---- ------- ------- ------- -------

1 31.99 31.99 13.21 13.21

2 11.18 43.18 14.08 27.29

3 4.68 47.86 24.57 51.86

4 5.99 53.84 10.56 62.42
