# Supplementary material for: A Systems Biology Strategy Reveals Biological Pathways and Plasma Biomarker Candidates for Potentially Toxic Statin-Induced Changes in Muscle
Source: PLoS One. 2006 Dec 20;1(1):e97. doi: 10.1371/journal.pone.0000097 (PMC1762369; doi:10.1371/journal.pone.0000097)
Supplement: Text S4 — Combined plasma lipidomics and muscle gene expression PLS/DA model details, corresponding to the Figure 2 of the paper. (0.03 MB DOC) [file pone.0000097.s004.doc]

X-block: X 17 by 170

Included: [ 6-10 17-22 29-34 ] [ 1-170 ]

Preprocessing: Autoscale

Y-block: y 17 by 3

Included: [ 6-10 17-22 29-34 ] [ 1-3 ]

Preprocessing: Autoscale

Num. LVs: 3

Cross validation: venetian blinds w/ 5 splits

RMSEC: 0 0 0

RMSECV: 0.24167 0.26515 0.29545

Percent Variance Captured by Regression Model

-----X-Block----- -----Y-Block-----

Comp This Total This Total

---- ------- ------- ------- -------

1 27.75 27.75 30.55 30.55

2 8.99 36.74 39.53 70.07

3 13.35 50.09 10.93 81.00

Q^2^=0.50
