# Supplementary material for: A Systems Biology Strategy Reveals Biological Pathways and Plasma Biomarker Candidates for Potentially Toxic Statin-Induced Changes in Muscle
Source: PLoS One. 2006 Dec 20;1(1):e97. doi: 10.1371/journal.pone.0000097 (PMC1762369; doi:10.1371/journal.pone.0000097)
Supplement: Table S1 — Characteristics of the patients selected for tissue gene expression analysis at baseline and at the end of the study. (0.03 MB DOC) [file pone.0000097.s005.doc]

Simvastatin (n=6) Atorvastatin (n=6) Placebo (n=6)

Baseline End Baseline End Baseline End

#### Age (years) 58.3 ± 6.3 58.7 ± 3.8 54.0 ± 10.5

#### BMI (kg/m^2^) 28.6 ± 2.1 27.4 ± 6.7 24.5 ± 1.5

#### S-Chol (mg/dL) 222.9 ± 35.1 152.5 ± 25.7 220.6 ± 17.5 148.8 ± 39.1 250.5 ± 35.2 251.7 ± 36.2

M-Chol (mg/g) 4.3 ± 0.6 6.9 ± 2.5 4.6 ± 1.3 5.5 ± 1.4 4.3 ± 1.4 4.8 ± 1.7

S-Ubi (μg/dL) 0.88 ± 0.24 0.55 ± 0.24 0.98 ± 0.20 0.69 ± 0.24 0.98 ± 0.19 1.0 ± 0.34

#### M-Ubi (μg/g) 36.2 ± 12.0 22.0 ± 5.8 * 40.3 ± 9.9 42.8 ± 19.1 46.0 ± 11.0 39.5 ± 18.2

Results are mean ± SD. *= p<0.05, for baseline vs. treatment comparison within group (paired t-test).
