# Supplementary material for: A Systems Biology Strategy Reveals Biological Pathways and Plasma Biomarker Candidates for Potentially Toxic Statin-Induced Changes in Muscle
Source: PLoS One. 2006 Dec 20;1(1):e97. doi: 10.1371/journal.pone.0000097 (PMC1762369; doi:10.1371/journal.pone.0000097)
Supplement: Table S2 — The top loads from the lipidomics analysis, ranked by increasing first latent variable (differentiating between the placebo and statin-treated groups as shown in Figure 1). LV1 therefore describes the lipid changes common to both statins. Negative LV1 values correspond to upregulation in placebo group. As expected the most abundant triacylglycerol, cholesterol ester, and phospholipids species are downregulated following the statin treatment. (0.05 MB DOC) [file pone.0000097.s006.doc]

| **ID** | **LV1** | **LV3** |
| --- | --- | --- |
| TG(50:0) | -0.17206 | -0.02828 |
| TG(50:1) | -0.16466 | -0.0093 |
| TG(48:1) | -0.16077 | -0.01345 |
| Cer(d18:1/22:0) | -0.1573 | -0.06337 |
| GPCho(32:0) | -0.15549 | 0.06558 |
| TG(50:0) | -0.1536 | -0.0336 |
| TG(48:0) | -0.15217 | -0.00383 |
| TG(52:1) | -0.14921 | -0.0884 |
| GPEtn(O-38:1) | -0.14531 | -0.03149 |
| ChoE(18:1) | -0.14491 | 0.13406 |
| GPCho(32:1) | -0.1415 | 0.02797 |
| TG(46:0) | -0.13886 | 0.00121 |
| GPCho(34:2) | -0.13826 | 0.05798 |
| GPCho(34:3) | -0.13738 | -0.01789 |
| GPCho(40:4) | -0.13587 | 0.04414 |
| TG(48:0) | -0.13499 | -0.0266 |
| TG(52:0) | -0.13293 | -0.08903 |
| GPCho(36:3) | -0.13282 | -0.08427 |
| SM(d18:1/16:0) | -0.1318 | 0.12223 |
| GPEtn(36:1) | -0.1314 | -0.09884 |
