# Supplementary material for: A Systems Biology Strategy Reveals Biological Pathways and Plasma Biomarker Candidates for Potentially Toxic Statin-Induced Changes in Muscle
Source: PLoS One. 2006 Dec 20;1(1):e97. doi: 10.1371/journal.pone.0000097 (PMC1762369; doi:10.1371/journal.pone.0000097)
Supplement: Table S3 — Lasso regression of plasma lipids on muscle ALOX5AP expression for NZ = 5 lipid variables. Lipid identifiers and their regression coefficients are listed. (0.04 MB DOC) [file pone.0000097.s007.doc]

| **ID** | **Lasso Coef** |
| --- | --- |
| GPEtn(38:4) | 5313.259 |
| GPEtn(40:4) | 188.5646 |
| GPEtn(42:6) | 3915.681 |
| ChoE(18:0) | -5113.89 |
| TG(52:3) | -92.0413 |
