# Supplementary material for: A Systems Biology Strategy Reveals Biological Pathways and Plasma Biomarker Candidates for Potentially Toxic Statin-Induced Changes in Muscle
Source: PLoS One. 2006 Dec 20;1(1):e97. doi: 10.1371/journal.pone.0000097 (PMC1762369; doi:10.1371/journal.pone.0000097)
Supplement: Table S4 — Lasso regression of plasma lipids on muscle ALOX5AP expression for NZ = 10 lipid variables. Lipid identifiers and their regression coefficients are listed. (0.04 MB DOC) [file pone.0000097.s008.doc]

| **ID** | **Lasso Coef** |
| --- | --- |
| GPCho(16:0/0:0) | -154.274 |
| GPCho(36:4) | 83.99264 |
| SM(d18:1/24:0) | -248.279 |
| SM(d18:1/24:1) | -31.9074 |
| GPEtn(38:4) | 5477.775 |
| GPEtn(42:6) | 4632.944 |
| ChoE(18:0) | -6320.96 |
| TG(51:2) | 252.9617 |
| TG(52:3) | -130.608 |
| TG(56:5) | 576.2522 |
