# Supplementary material for: A Systems Biology Strategy Reveals Biological Pathways and Plasma Biomarker Candidates for Potentially Toxic Statin-Induced Changes in Muscle
Source: PLoS One. 2006 Dec 20;1(1):e97. doi: 10.1371/journal.pone.0000097 (PMC1762369; doi:10.1371/journal.pone.0000097)
Supplement: Table S5 — Lasso regression of plasma lipids on muscle ALOX5AP expression for NZ = 15 lipid variables. Lipid identifiers and their regression coefficients are listed. (0.04 MB DOC) [file pone.0000097.s009.doc]

| **ID** | **Lasso Coef** |
| --- | --- |
| GPCho(16:0/0:0) | -567.526 |
| GPCho(36:4) | 105.3319 |
| GPCho(36:4) | 1262.811 |
| GPCho(38:5) | -645.201 |
| GPCho(O-36:2) | 4110.83 |
| SM(d18:1/24:1) | -402.198 |
| GPEtn(36:2) | 686.9336 |
| GPEtn(38:4) | 4234.713 |
| GPEtn(42:6) | 4368.903 |
| ChoE(18:0) | -7225.02 |
| TG(51:2) | 1139.847 |
| TG(51:3) | 658.5384 |
| TG(52:3) | -232.222 |
| TG(54:3) | 62.39275 |
| TG(56:5) | 1156.534 |
