# Supplementary material for: A Systems Biology Strategy Reveals Biological Pathways and Plasma Biomarker Candidates for Potentially Toxic Statin-Induced Changes in Muscle
Source: PLoS One. 2006 Dec 20;1(1):e97. doi: 10.1371/journal.pone.0000097 (PMC1762369; doi:10.1371/journal.pone.0000097)
Supplement: Table S6 — Lasso regression of plasma lipids on muscle ALOX5AP expression for NZ = 20 lipid variables. Lipid identifiers and their regression coefficients are listed. (0.04 MB DOC) [file pone.0000097.s010.doc]

| **ID** | **Lasso Coef** |
| --- | --- |
| GPCho(18:0/0:0) | 240.789 |
| GPCho(36:4) | 233.1943 |
| GPCho(36:4) | 7156.164 |
| GPCho(36:5) | -1260.5 |
| GPCho(38:3) | 1342.655 |
| GPCho(38:5) | -2914.57 |
| GPCho(38:7) | -7507.23 |
| GPCho(O-38:5) | -7397.49 |
| SM(d18:1/24:0) | -1872.93 |
| SM(d18:1/24:1) | -455.065 |
| GPEtn(36:2) | 2514.609 |
| GPEtn(38:1) | 179.5402 |
| GPEtn(38:4) | 991.0704 |
| GPEtn(42:6) | 4241.529 |
| ChoE(18:0) | -7692.19 |
| TG(51:1) | 634.5522 |
| TG(51:2) | 1912.152 |
| TG(52:3) | -336.489 |
| TG(54:3) | 45.95206 |
| TG(56:5) | 3404.53 |
