# Supplementary material for: A Systems Biology Strategy Reveals Biological Pathways and Plasma Biomarker Candidates for Potentially Toxic Statin-Induced Changes in Muscle
Source: PLoS One. 2006 Dec 20;1(1):e97. doi: 10.1371/journal.pone.0000097 (PMC1762369; doi:10.1371/journal.pone.0000097)
Supplement: Table S7 — Characteristics of the lasso regression (plasma lipids on muscle ALOX5AP expression) models for different number of non-zero regression coefficients. (0.04 MB DOC) [file pone.0000097.s011.doc]

| **NZ** | **corrected R^2** | **SC*** | **corr (pearson)** | **p-val (corr = 0)** |
| --- | --- | --- | --- | --- |
| 25 | 0.909767 | 10.32333 | 0.992121 | 0 |
| 20 | 0.769197 | 11.18575 | 0.970391 | 0 |
| 15 | 0.481729 | 11.78149 | 0.893821 | 1.11E-12 |
| 10 | 0.324289 | 11.76179 | 0.785987 | 3.63E-08 |
| 5 | 0.327381 | 11.42786 | 0.73388 | 7.86E-07 |

*SC=Schwartz criterion
