# Supplementary material for: A Systems Biology Strategy Reveals Biological Pathways and Plasma Biomarker Candidates for Potentially Toxic Statin-Induced Changes in Muscle
Source: PLoS One. 2006 Dec 20;1(1):e97. doi: 10.1371/journal.pone.0000097 (PMC1762369; doi:10.1371/journal.pone.0000097)
Supplement: Table S8 — Standard compounds used in lipidomics platform, their monoisotopic masses, the fragments used and average retention times. (0.06 MB DOC) [file pone.0000097.s012.doc]

| Compound | Monoisotopic mass (m/z) | Detected | Retention time (s) |
| --- | --- | --- | --- |
| D-*erythro*-Sphingosine-1-Phosphate | 365.23311 | **M-115**, M+1, M-17 | 137.4 |
| GPCho(16:0/0:0-D3) | 498.34688 | **M+1** | 196.2 |
| GPCho(17:0/0:0) | 509.34814 | **M+1** | 217.2 |
| MG(17:0/0:0/0:0) [rac] | 344.29266 | **M+23**, M+1, M-17 | 265.8 |
| GPCho(16:0/16:0-D6) | 735.59094 | **M+1** | 378.0 |
| GPGro(17:0/17:0)[rac] | 750.54109 | **M+1** | 385.2 |
| Cer(d18:1/17:0) | 551.52774 | **M+1** | 385.8 |
| GPSer(17:0/17:0) | 763.53634 | **M+1** | 390.6 |
| GPCho(17:0/17:0) | 761.59346 | **M+1** | 393.6 |
| GPA(17:0/17:0) | 676.50431 | **M+18**, M+1 | 394.8 |
| GPEtn(17:0/17:0) | 719.54651 | **M+1** | 399.0 |
| DG(17:0/17:0/0:0) [rac] | 596.53798 | **M+18**, M+1, M-17 | 411.6 |
| TG(16:0/16:0/16:0-13C3) | 809.77640 | **M+18** | 497.4 |
| TG(17:0/17:0/17:0) | 848.78329 | **M+18** | 543.0 |

Most intense ions marked in bold.
