# Supplementary material for: A Systems Biology Strategy Reveals Biological Pathways and Plasma Biomarker Candidates for Potentially Toxic Statin-Induced Changes in Muscle
Source: PLoS One. 2006 Dec 20;1(1):e97. doi: 10.1371/journal.pone.0000097 (PMC1762369; doi:10.1371/journal.pone.0000097)
Supplement: Table S9 — Recovery data for three internal lipid standards. (0.05 MB DOC) [file pone.0000097.s013.doc]

| Lipid compound | Added amount (µg/ml) | Lipid /  Labeled lipid standard | Recovery (%) |
| --- | --- | --- | --- |
|  |  | GPCho(17:0/0:0)/  GPCho(16:0/0:0-D3) |  |
| GPCho(17:0/0:0) | 32,04 | 3,46 | 100,1 |
|  | 64,08 | 6,71 | 97,2 |
|  | 96,12 | 10,29 | 99,3 |
|  | 160,2 | 15,85 | 91,8 |
|  |  | GPCho(17:0/17:0)/  GPCho(16:0/16:0-D6) |  |
| GPCho(17:0/17:0) | 0,99 | 0,078 | 92,0 |
|  | 1,98 | 0,161 | 94,9 |
|  | 2,97 | 0,245 | 96,3 |
|  | 4,95 | 0,400 | 94,4 |
|  |  | TG(17:0/17:0/17:0)/  TG(16:0/16:0/16:0-13C3) |  |
| TG(17:0/17:0/17:0) | 10,36 | 1,03 | 99,3 |
|  | 20,72 | 1,97 | 94,9 |
|  | 31,08 | 2,91 | 93,4 |
|  | 51,8 | 3,52 | 67,7 |
