# Supplementary material for: A Systems Biology Strategy Reveals Biological Pathways and Plasma Biomarker Candidates for Potentially Toxic Statin-Induced Changes in Muscle
Source: PLoS One. 2006 Dec 20;1(1):e97. doi: 10.1371/journal.pone.0000097 (PMC1762369; doi:10.1371/journal.pone.0000097)
Supplement: Table S10 — Repeatability of the UPLC/MS runs determined from 10 successive injections, as determined from the same liver extract. (0.05 MB DOC) [file pone.0000097.s014.doc]

**Absolute peak heights**

| Lipid compound | Mean (n=10) | SD | CV% |
| --- | --- | --- | --- |
| GPCho(16:0/0:0-D3) | 2579 | 169 | 6,56 |
| GPCho(17:0/0:0) | 17572 | 1048 | 5,96 |
| GPEtn(17:0/17:0) | 1214 | 86 | 7,07 |
| GPCho(16:0/16:0-D6) | 1773 | 70 | 3,97 |
| GPCho(17:0/17:0) | 1902 | 104 | 5,49 |
| TG(16:0/16:0/16:0-13C3) | 377 | 18 | 4,84 |
| TG(17:0/17:0/17:0) | 511 | 44 | 8,60 |
| Cer(d18:1/17:0) | 44 | 3 | 7,53 |

**Comparison to a labeled standard**

| Lipid compound /  Labeled standard | Mean (n=10) | SD | CV% |
| --- | --- | --- | --- |
| GPCho(17:0/0:0)/ GPCho(16:0/0:0-D3) | 6,845 | 0,6500 | 9,43 |
| GPEtn(17:0/17:0)/ GPCho(16:0/16:0-D6) | 0,684 | 0,0300 | 4,67 |
| GPCho(17:0/17:0)/ GPCho(16:0/16:0-D6) | 1,075 | 0,0800 | 7,86 |
| TG(17:0/17:0/17:0)/ TG(16:0/16:0/16:0-13C3) | 1,357 | 0,1100 | 7,99 |
| Cer(d18:1/17:0)/ GPCho(16:0/16:0-D6) | 0,025 | 0,0015 | 5,91 |
