# Supplementary material for: A Systems Biology Strategy Reveals Biological Pathways and Plasma Biomarker Candidates for Potentially Toxic Statin-Induced Changes in Muscle
Source: PLoS One. 2006 Dec 20;1(1):e97. doi: 10.1371/journal.pone.0000097 (PMC1762369; doi:10.1371/journal.pone.0000097)
Supplement: Table S11 — Repeatability of the analysis including standard addition, extraction and UPLC/MS analysis. (0.05 MB DOC) [file pone.0000097.s015.doc]

**Absolute peak heights.**

| Lipid compound | Mean (n=6) | SD | CV% |
| --- | --- | --- | --- |
| GPCho(16:0/0:0-D3) | 2756 | 284 | 10,29 |
| GPCho(17:0/0:0) | 19377 | 1789 | 9,23 |
| Cer(d18:1/17:0) | 70 | 9 | 12,19 |
| GPEtn(17:0/17:0) | 1399 | 170 | 12,15 |
| GPCho(16:0/16:0-D6) | 1930 | 64 | 3,31 |
| GPCho(17:0/17:0) | 1935 | 113 | 5,85 |
| TG(16:0/16:0/16:0-13C3) | 536 | 46 | 8,66 |
| TG(17:0/17:0/17:0) | 572 | 61 | 10,65 |

**In comparison to labeled standards.**

| Lipid standard /  labeled standard | Mean (n=6) | SD | CV% |
| --- | --- | --- | --- |
| GPCho(17:0/0:0)/ GPCho(16:0/0:0-D3) | 7,004 | 0,475 | 6,78 |
| Cer(d18:1/17:0)/ GPCho(16:0/16:0-D6) | 0,035 | 0,004 | 11,11 |
| GPEtn(17:0/17:0)/ GPCho(16:0/16:0-D6) | 0,729 | 0,074 | 10,18 |
| GPCho(17:0/17:0)/ GPCho(16:0/16:0-D6) | 1,004 | 0,062 | 6,22 |
| TG(17:0/17:0/17:0)/ TG(16:0/16:0/16:0-13C3) | 1,278 | 0,127 | 9,97 |
