# Supplementary figures and images for: A Systems Biology Strategy Reveals Biological Pathways and Plasma Biomarker Candidates for Potentially Toxic Statin-Induced Changes in Muscle
Source: PLoS One. 2006 Dec 20;1(1):e97. doi: 10.1371/journal.pone.0000097 (PMC1762369; doi:10.1371/journal.pone.0000097)

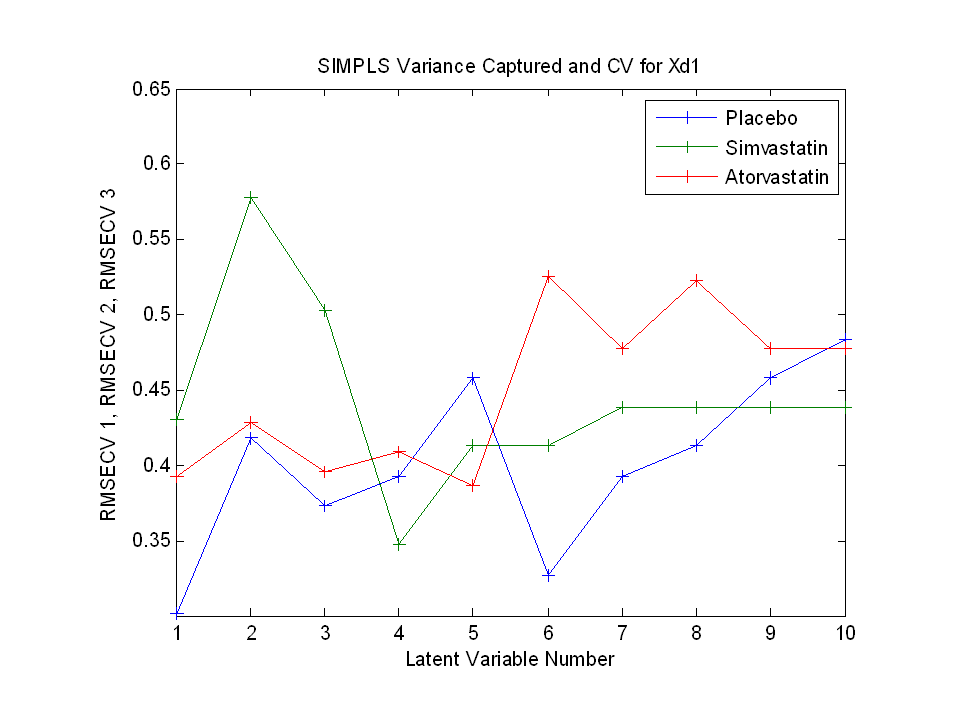

Supplement: Figure S1 — PLS/DA model for lipidomics analysis (corresponding to Figure 1). Root-Mean-Square Error of Cross-Validation (RMSECV) for three intervention groups. Four latent variables were chosen for the model. (0.07 MB TIF) [file pone.0000097.s016.tif]

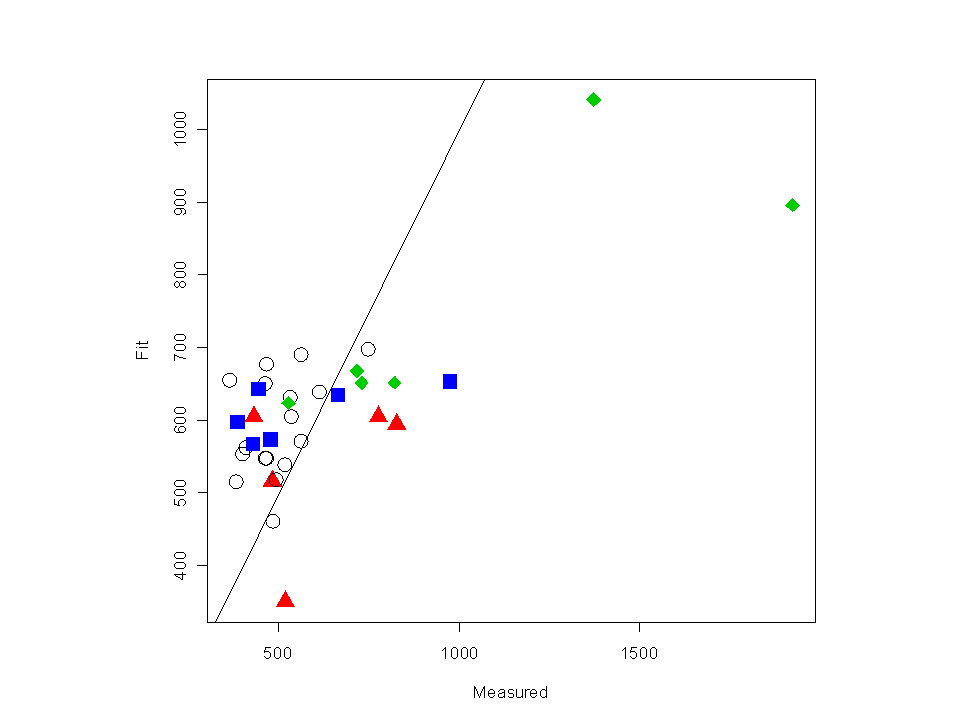

Supplement: Figure S2 — Lasso regression of plasma lipids on muscle ALOX5AP expression for NZ = 5 lipid variables. ALOX5AP expression values as predicted by the model. (0.05 MB TIF) [file pone.0000097.s017.tif]

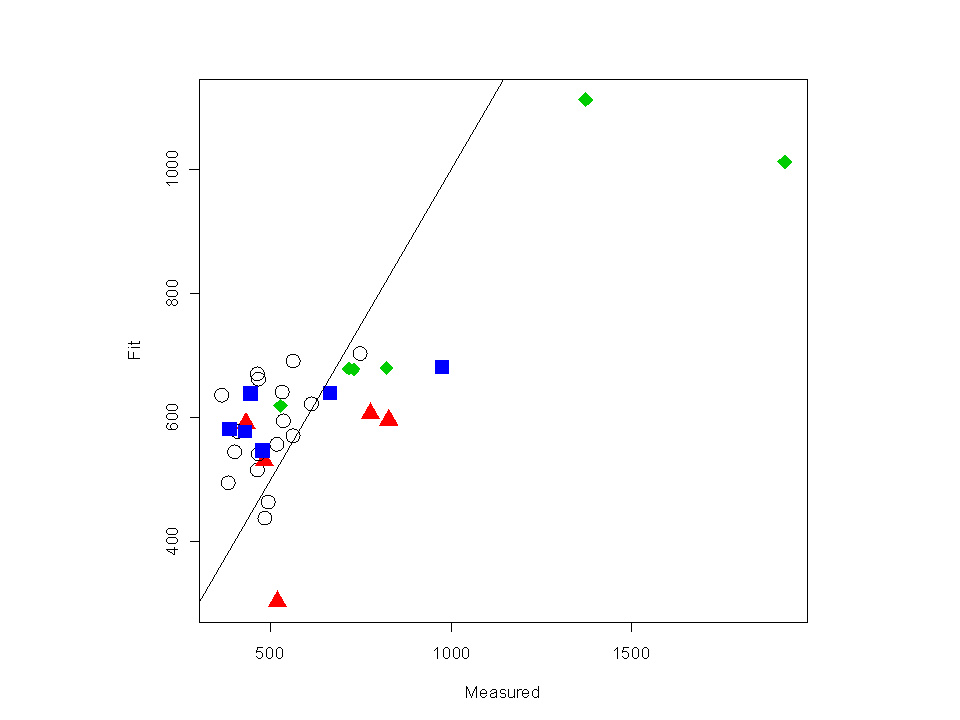

Supplement: Figure S3 — Lasso regression of plasma lipids on muscle ALOX5AP expression for NZ = 10 lipid variables. ALOX5AP expression values as predicted by the model. (0.05 MB TIF) [file pone.0000097.s018.tif]

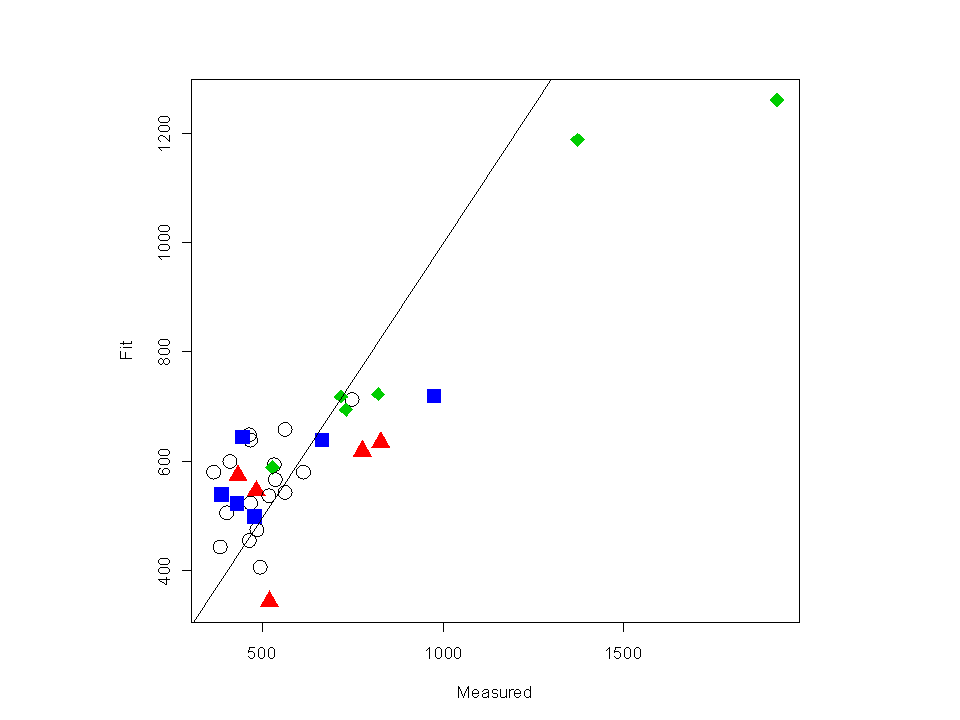

Supplement: Figure S4 — Lasso regression of plasma lipids on muscle ALOX5AP expression for NZ = 15 lipid variables. ALOX5AP expression values as predicted by the model. (0.05 MB TIF) [file pone.0000097.s019.tif]

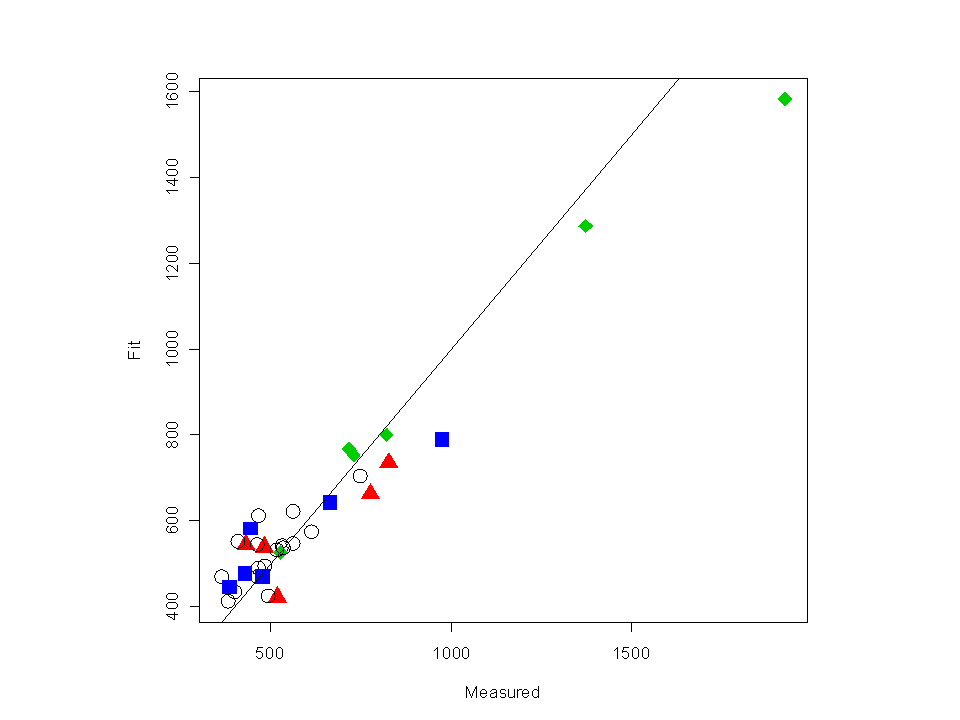

Supplement: Figure S5 — Lasso regression of plasma lipids on muscle ALOX5AP expression for NZ = 20 lipid variables. ALOX5AP expression values as predicted by the model. (0.05 MB TIF) [file pone.0000097.s020.tif]

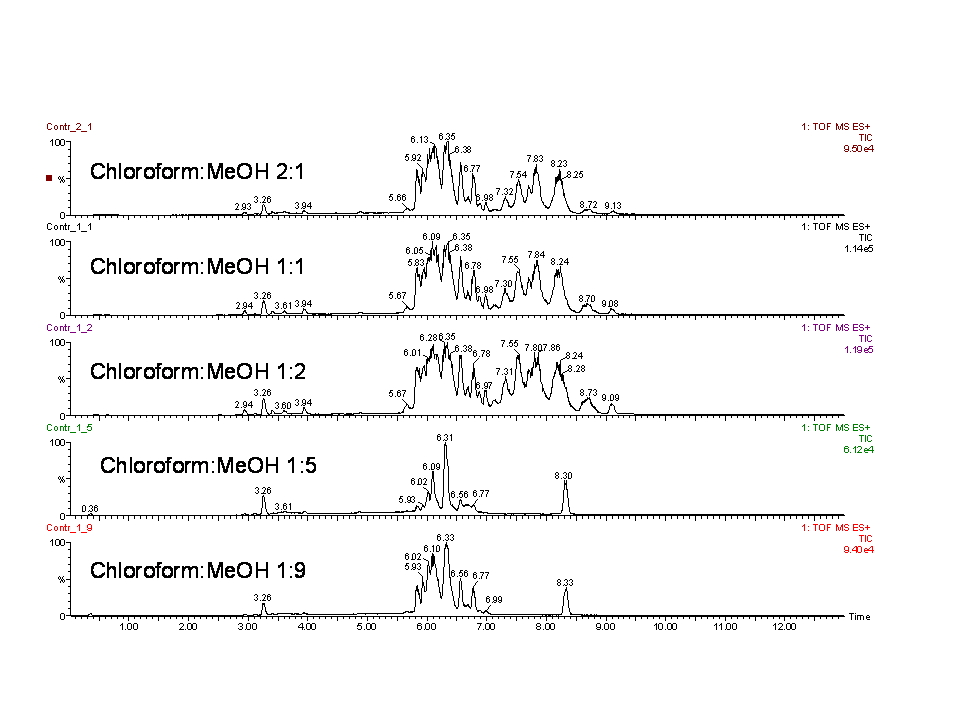

Supplement: Figure S6 — Total ion UPLC/MS chromatograms for different extractions of a control serum sample, with chloroform:methanol in ratios 1:9 - 2:1. (0.07 MB TIF) [file pone.0000097.s021.tif]

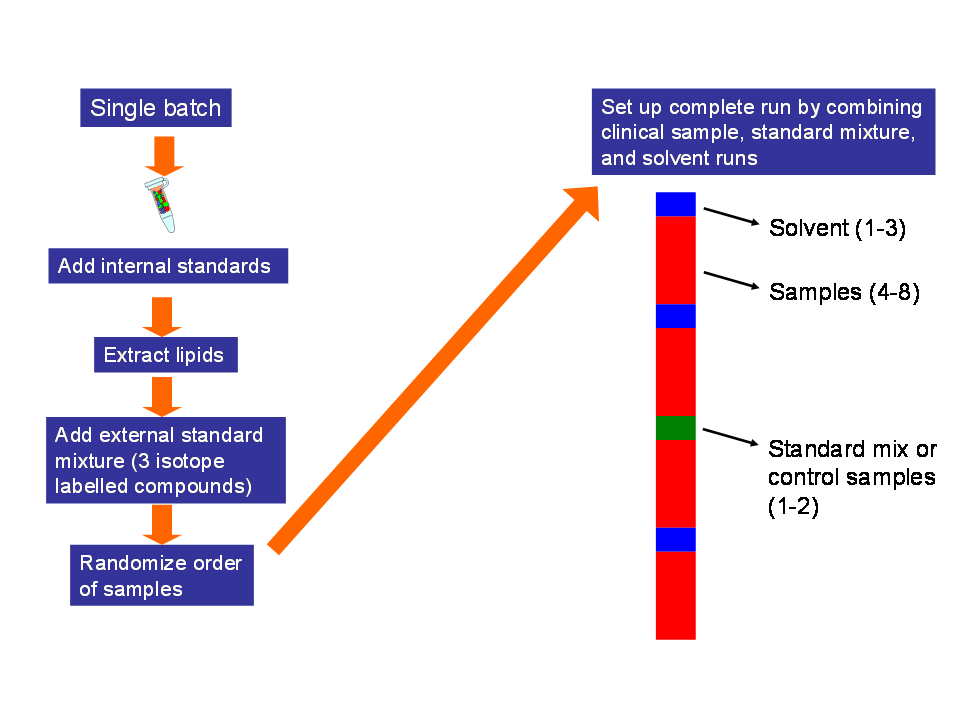

Supplement: Figure S7 — Setup of lipidomics runs. (0.11 MB TIF) [file pone.0000097.s022.tif]

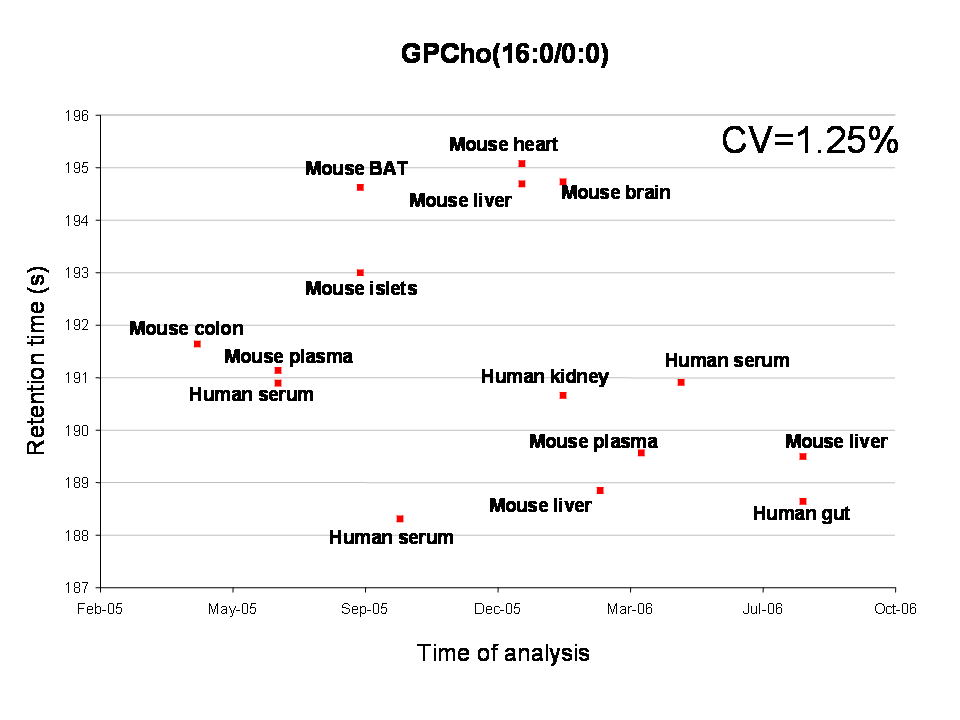

Supplement: Figure S8 — Variability of retention time for lysophosphatidylcholine GPCho(16:0/0:0) over a period of 18 months as determined from different experiments across different tissues and analytical UPLC C18 columns. (0.07 MB TIF) [file pone.0000097.s023.tif]

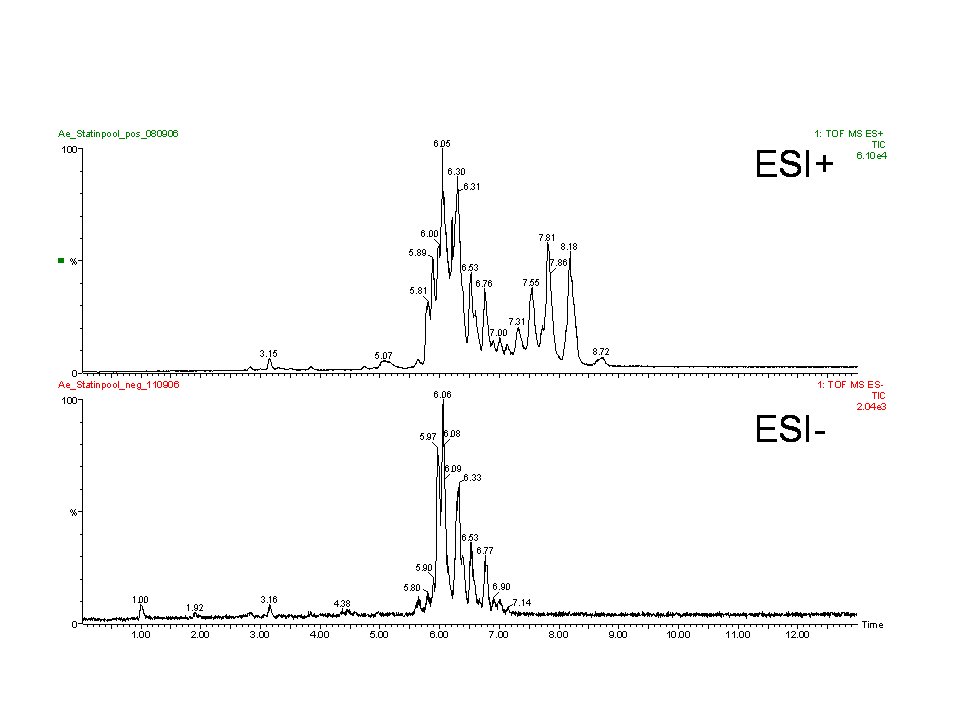

Supplement: Figure S9 — An example of typical UPLC/MS total ion chromatograms (TIC) from a serum lipid extract in ESI+ and ESI- mode. (0.06 MB TIF) [file pone.0000097.s024.tif]

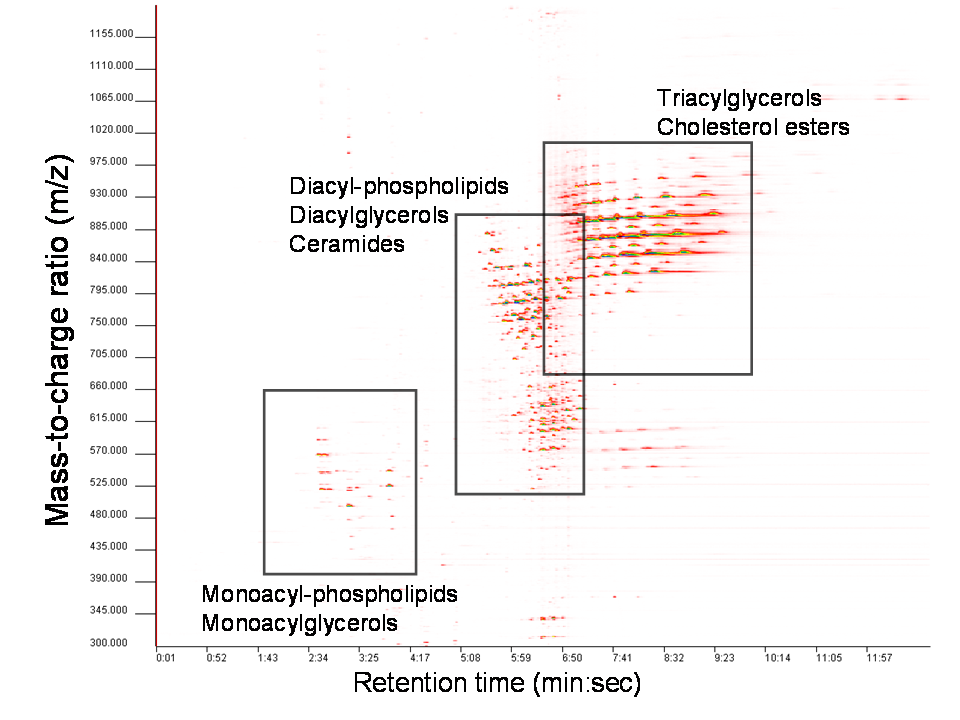

Supplement: Figure S10 — Two dimensional view of typical lipidomics spectra. (0.28 MB TIF) [file pone.0000097.s025.tif]

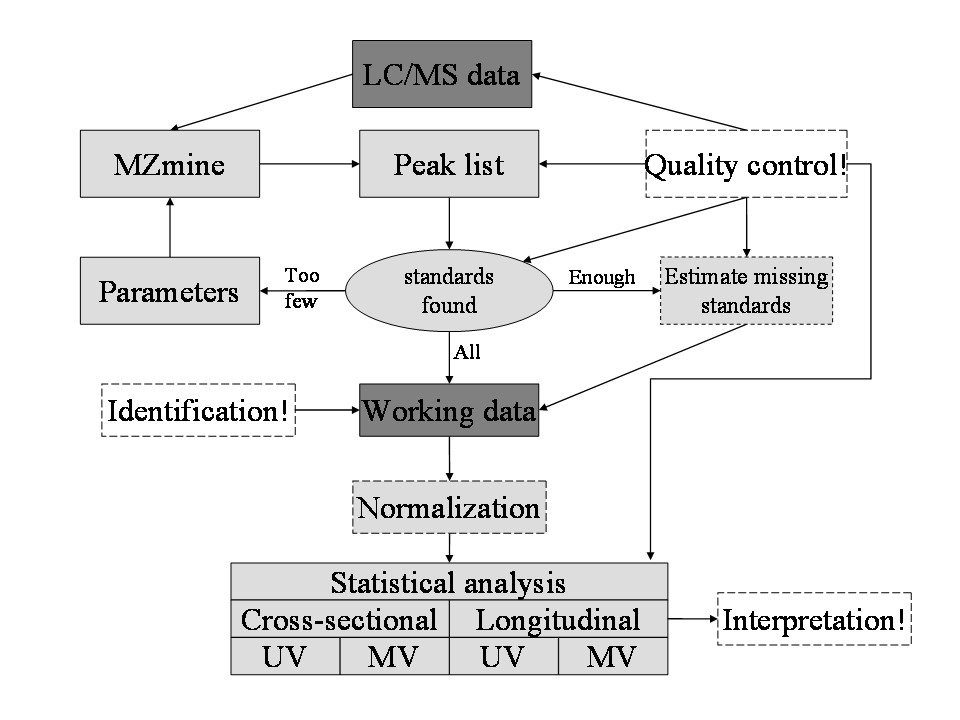

Supplement: Figure S11 — Quality control flow-chart for metabolomics data processing. (0.12 MB TIF) [file pone.0000097.s026.tif]

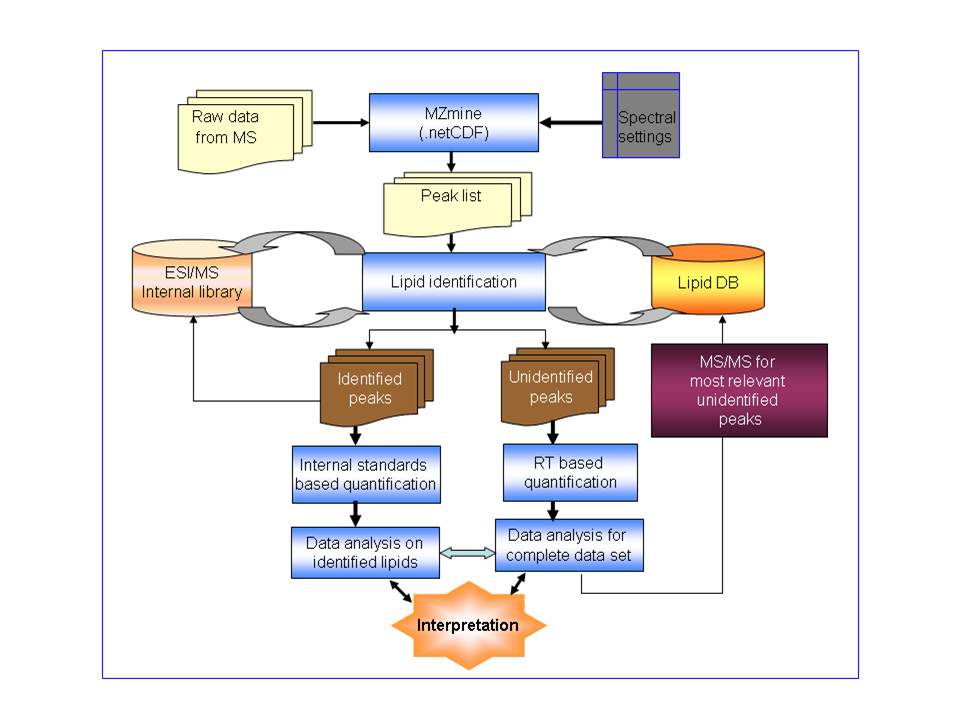

Supplement: Figure S12 — Lipidomics data processing and identification workflow. (0.31 MB TIF) [file pone.0000097.s027.tif]

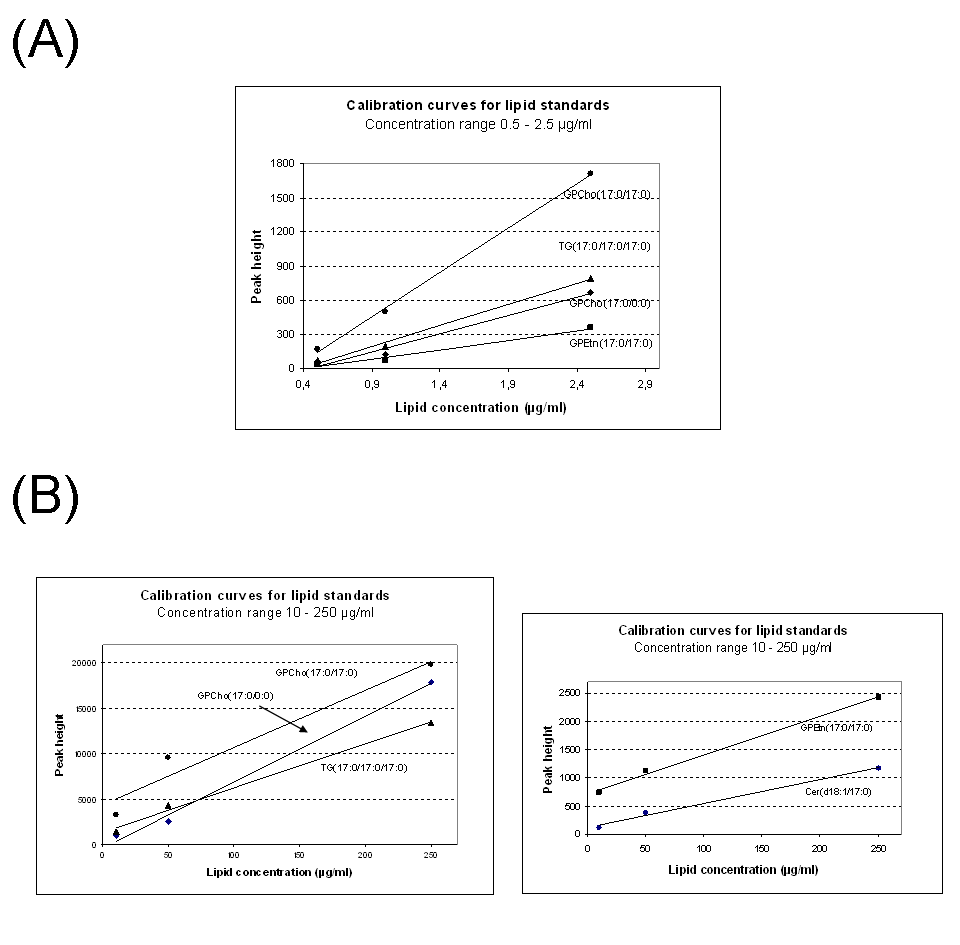

Supplement: Figure S13 — Calibration curves for internal lipid standards (added to samples prior to extraction) as determined from human serum total lipid extracts. (A) Concentration range 0.5-2.5 μg/ml. (B) Concentration range10-250 μg/ml. Lines drawn as guides. (0.09 MB TIF) [file pone.0000097.s028.tif]

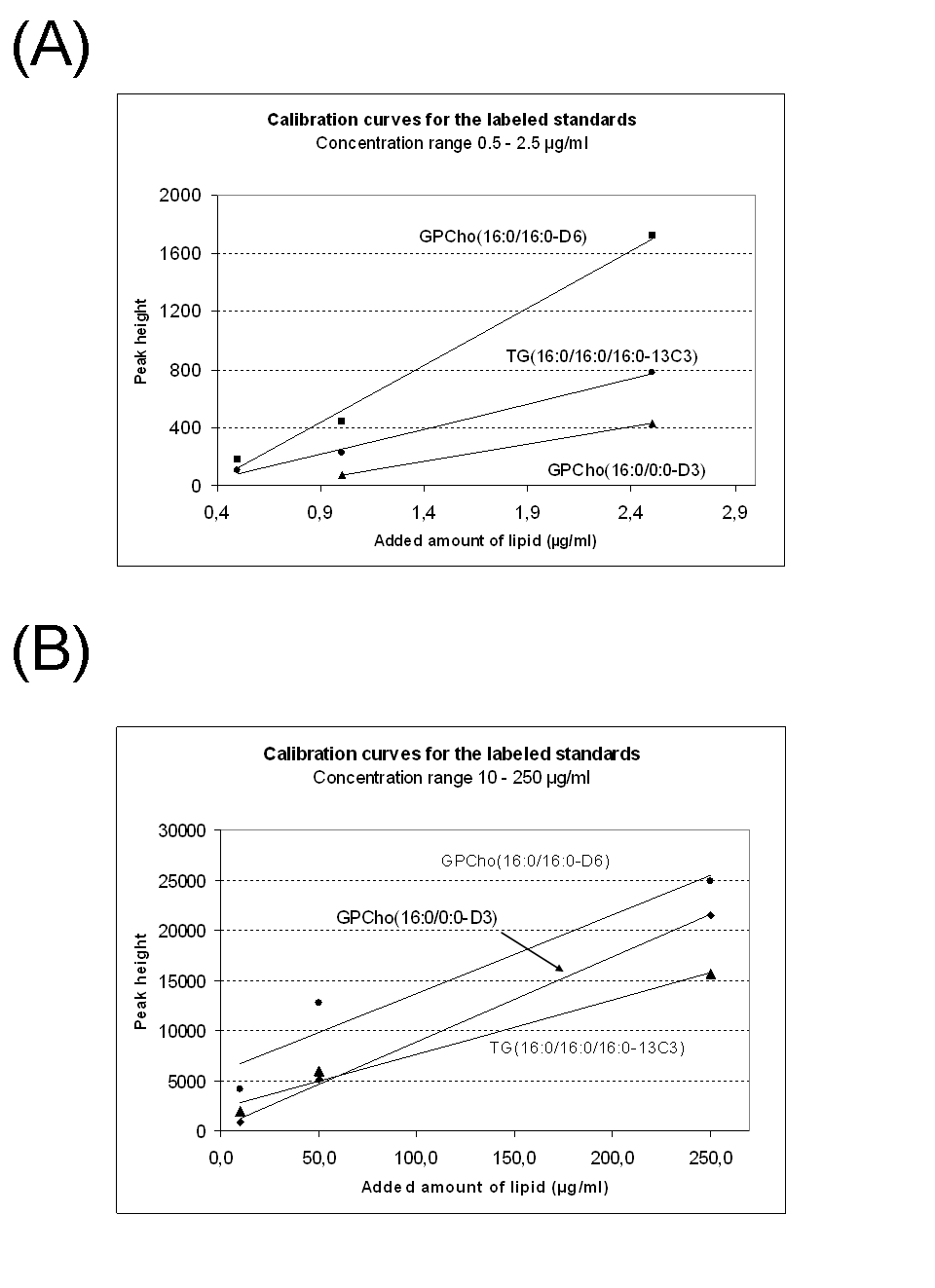

Supplement: Figure S14 — Calibration curves for labeled external lipid standards (added to samples after the extraction) as determined from human serum total lipid extracts. (A) Concentration range 0.5-2.5 μg/ml. (B) Concentration range10-250 μg/ml. (0.13 MB TIF) [file pone.0000097.s029.tif]

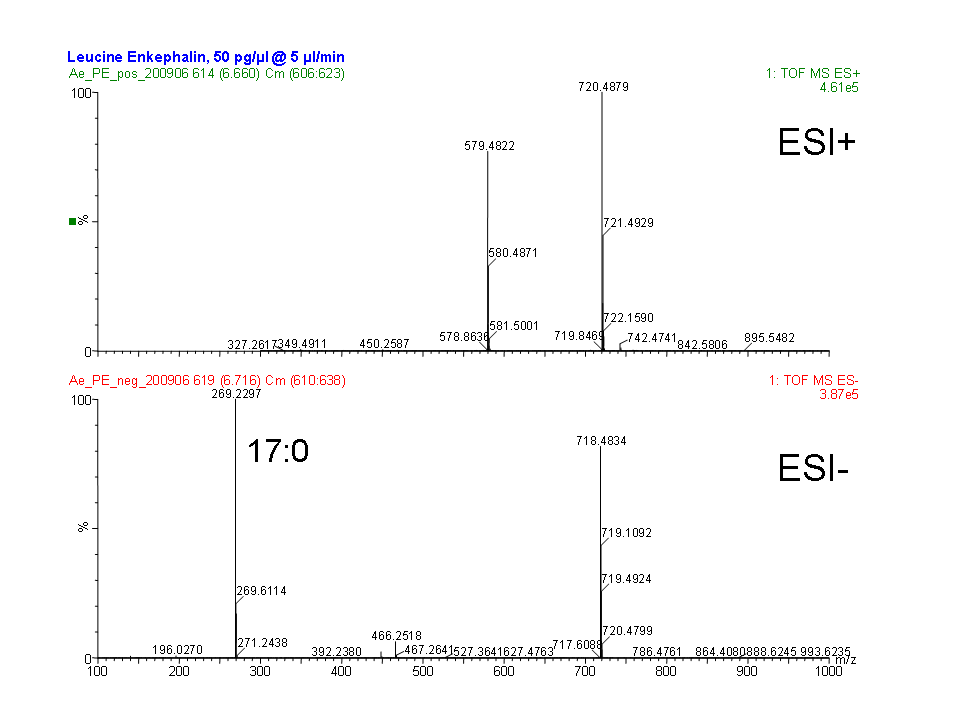

Supplement: Figure S15 — MS/MS spectra of phosphatidylethanolamine GPEtn(17:0/17:0) in ESI+ and ESI-. (0.07 MB TIF) [file pone.0000097.s030.tif]

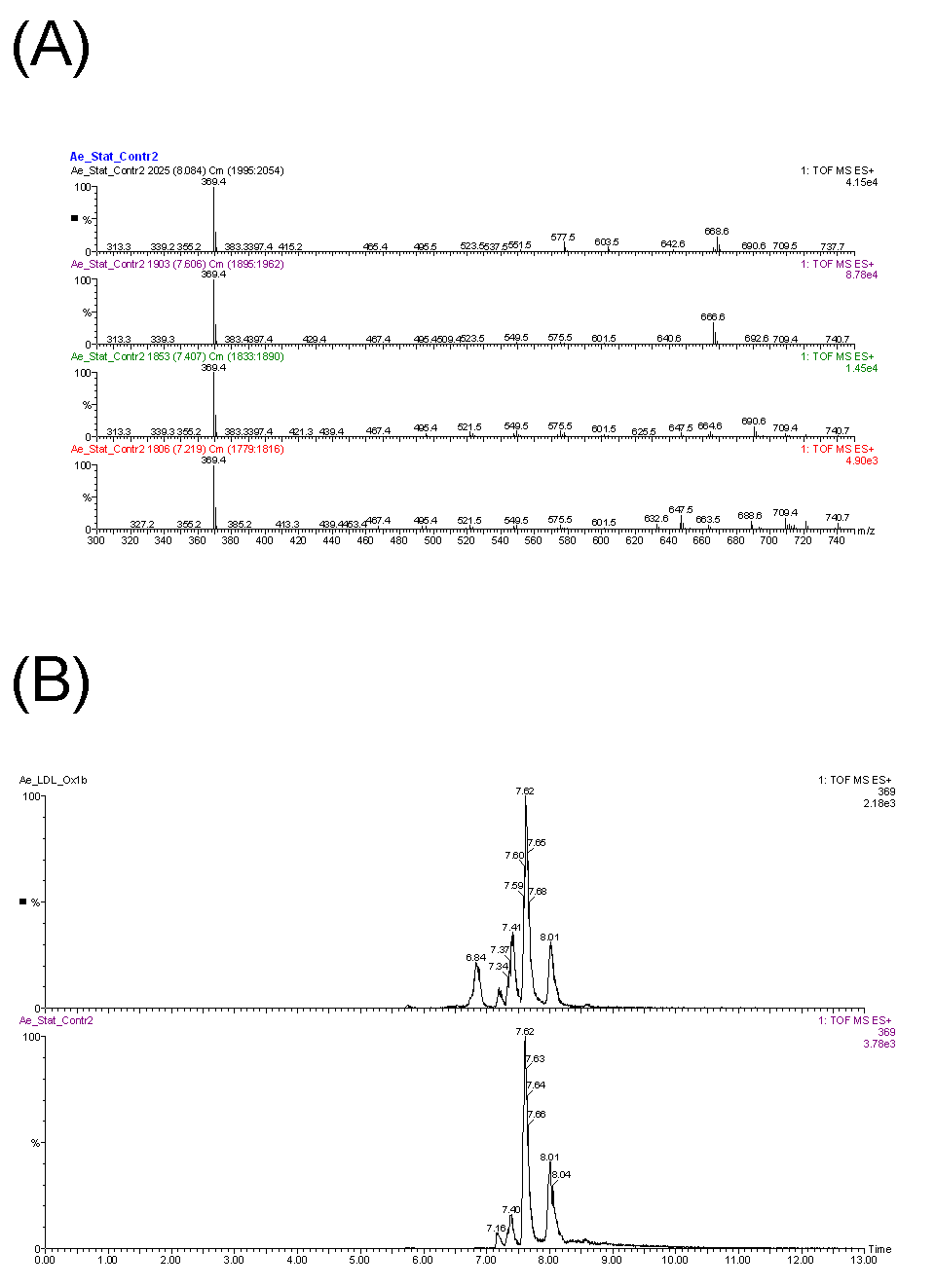

Supplement: Figure S16 — (A) Mass spectra of selected cholesteryl esters in human serum. (B) The extracted ion chromatogram of cholesteryl esters (as detected at m/z 369) in serum LDL-fraction and in total serum. (0.11 MB TIF) [file pone.0000097.s031.tif]

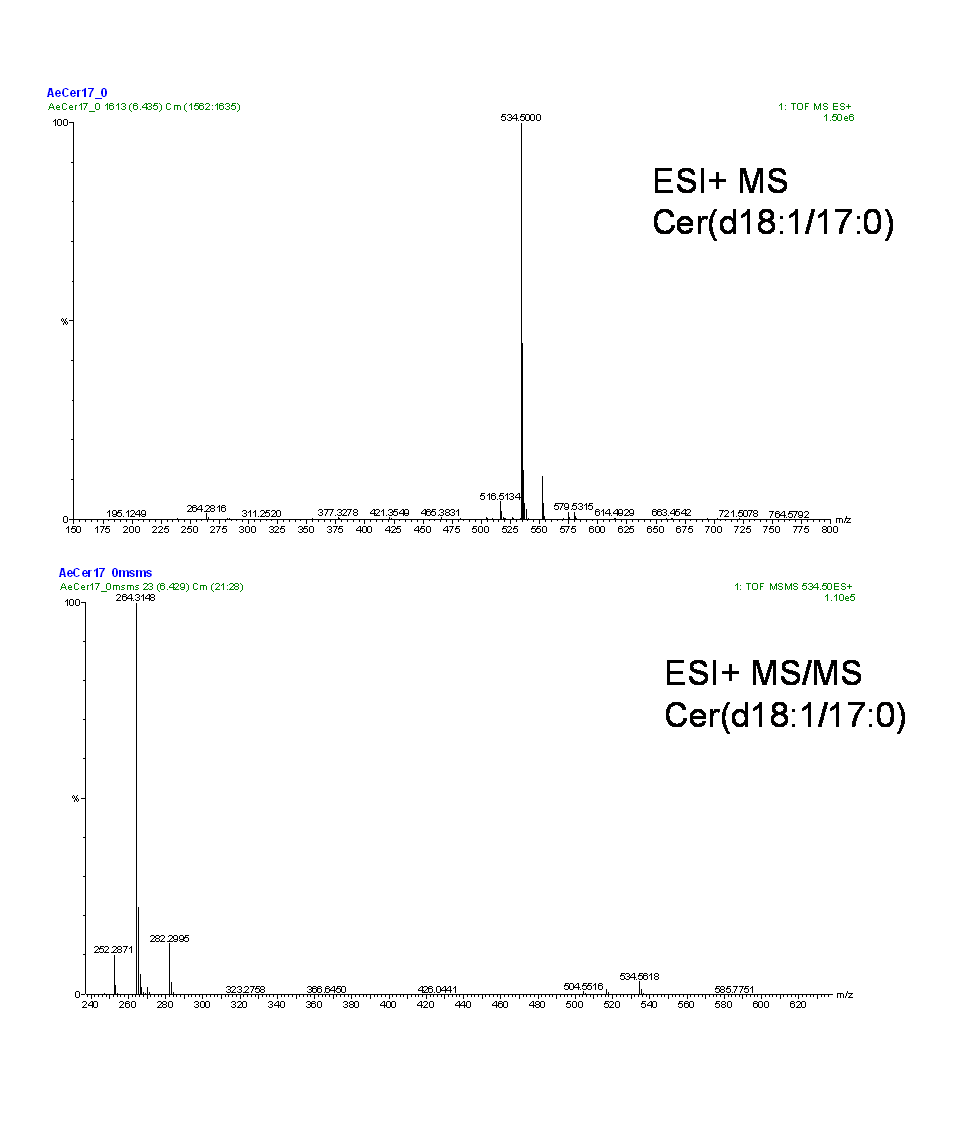

Supplement: Figure S17 — ESI+ MS and MS/MS spectra of ceramide Cer(d18:1/17:0). (0.09 MB TIF) [file pone.0000097.s032.tif]

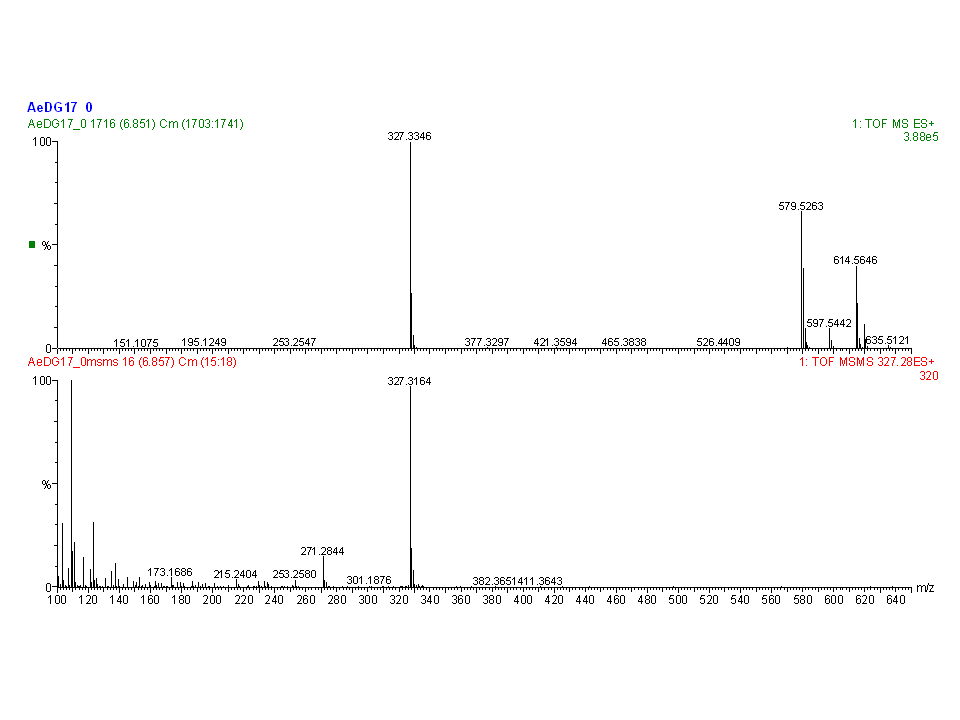

Supplement: Figure S18 — ESI+ MS and MS/MS spectrum of diacylglycerol DG(17:0/17:0). (0.06 MB TIF) [file pone.0000097.s033.tif]

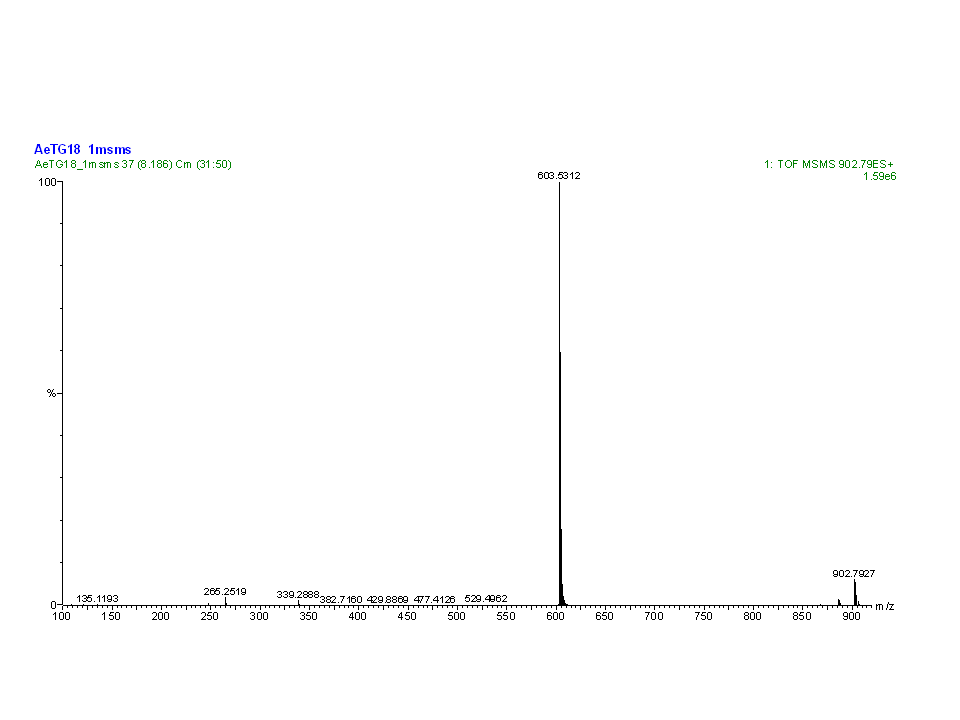

Supplement: Figure S19 — ESI+ MS/MS spectrum of triacylglycerol TG(17:0/17:0/17:0), with base peak m/z 903 and the m/z 603 peak corresponding to neutral loss of fatty acyl 17:0. (0.05 MB TIF) [file pone.0000097.s034.tif]

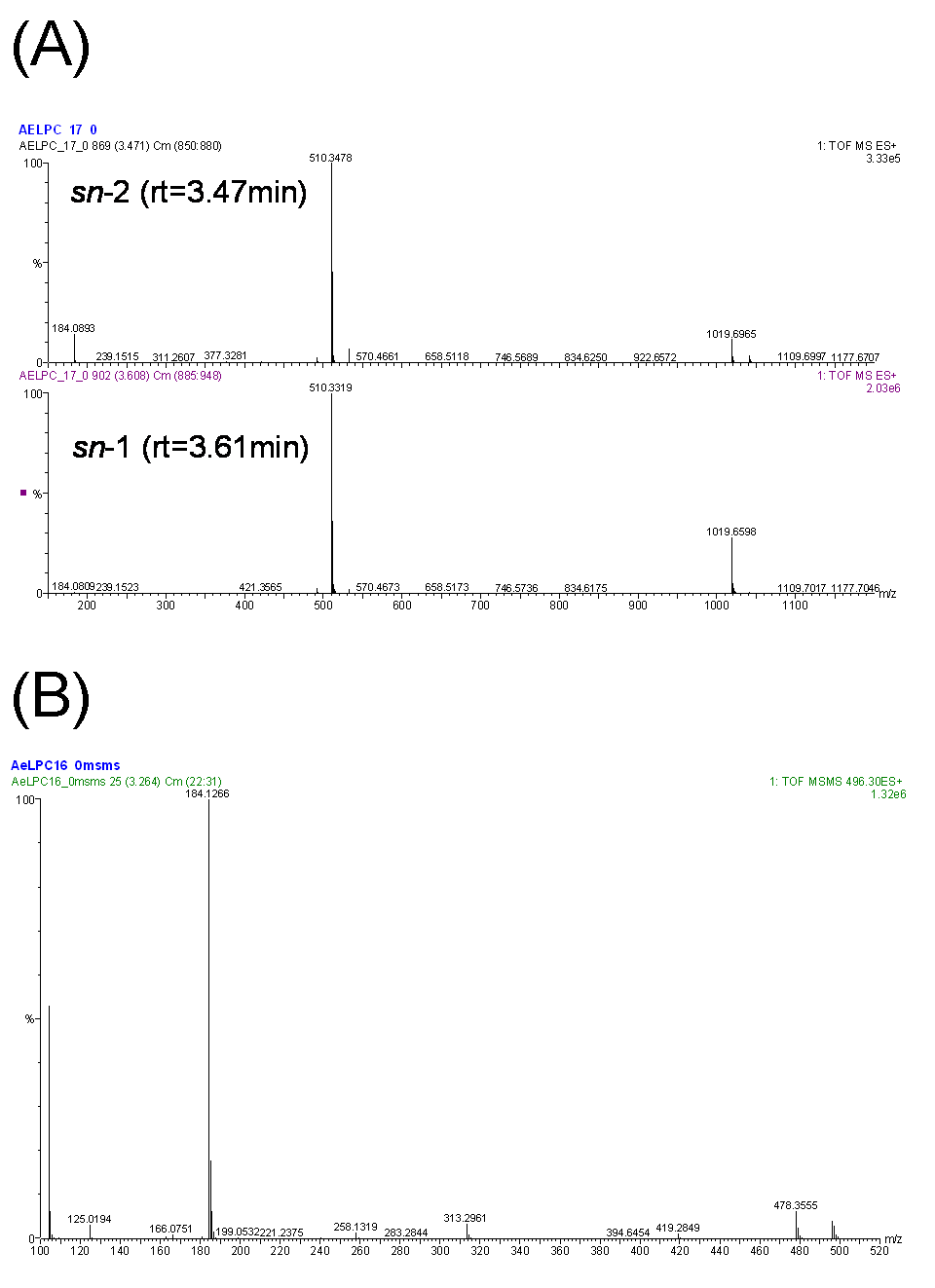

Supplement: Figure S20 — (A) Mass spectra of lysophosphatidylcholine GPCho(17:0/0:0), two separate chromatographic peaks in serum lipid extracts (sn-2 and sn-1). (B) ESI+ MS/MS spectrum of GPCho(16:0/0:0), with the characteristic m/z 184 peak. (0.11 MB TIF) [file pone.0000097.s035.tif]

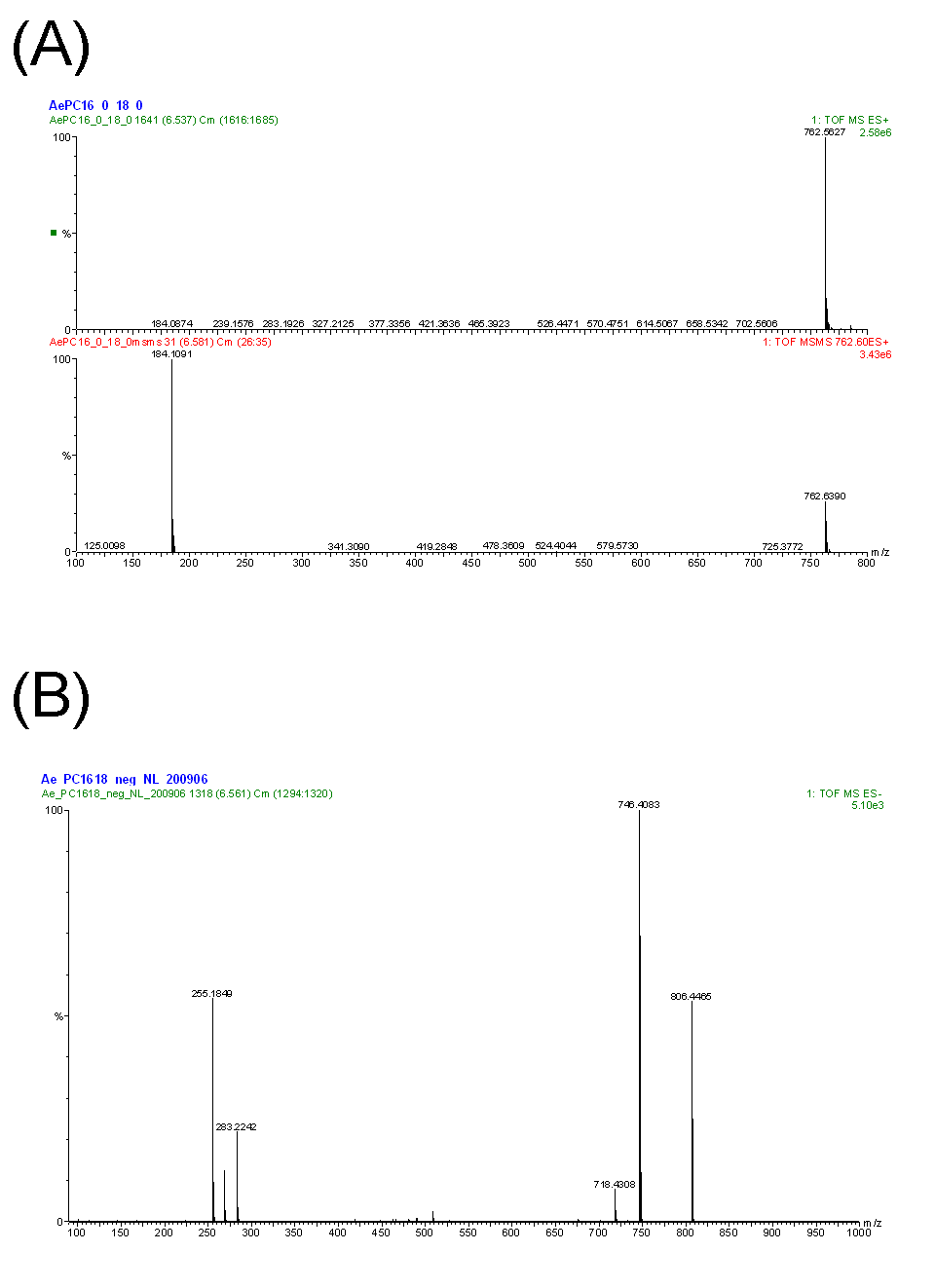

Supplement: Figure S21 — (A) ESI+ MS and MSMS spectra of GPCho(16:0/18:0). (B) ESI- MS spectrum of GPCho(16:0/18:0). (0.10 MB TIF) [file pone.0000097.s036.tif]

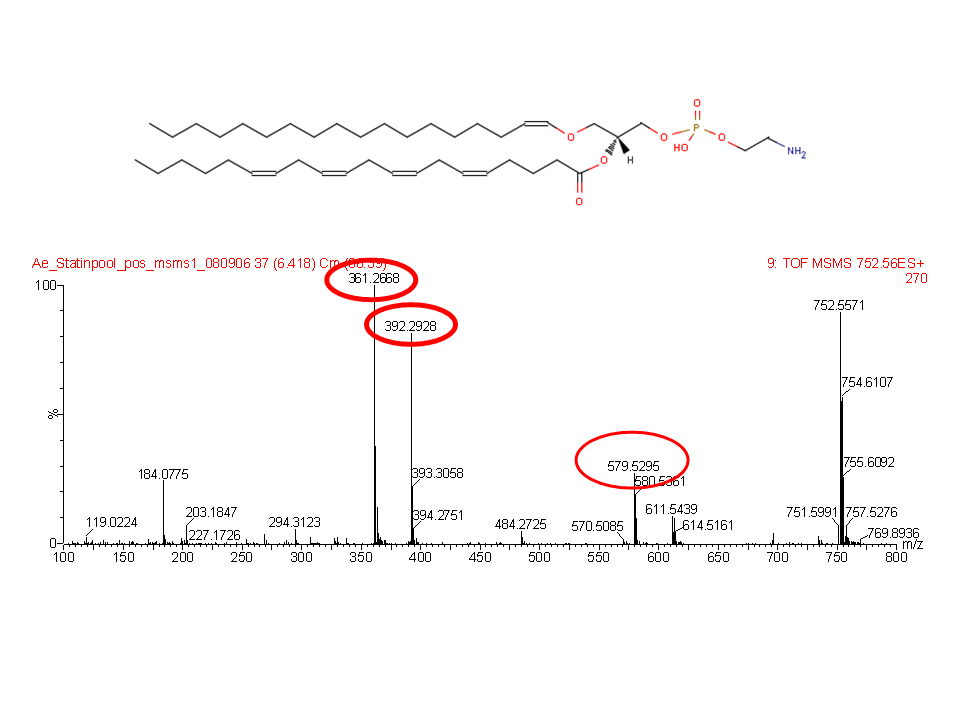

Supplement: Figure S22 — ESI+ MS/MS spectrum of the ethanolamine plasmalogen GPEtn(O-18:1(1Z)/20:4), with the characteristic m/z 361 and m/z 392. (0.07 MB TIF) [file pone.0000097.s037.tif]

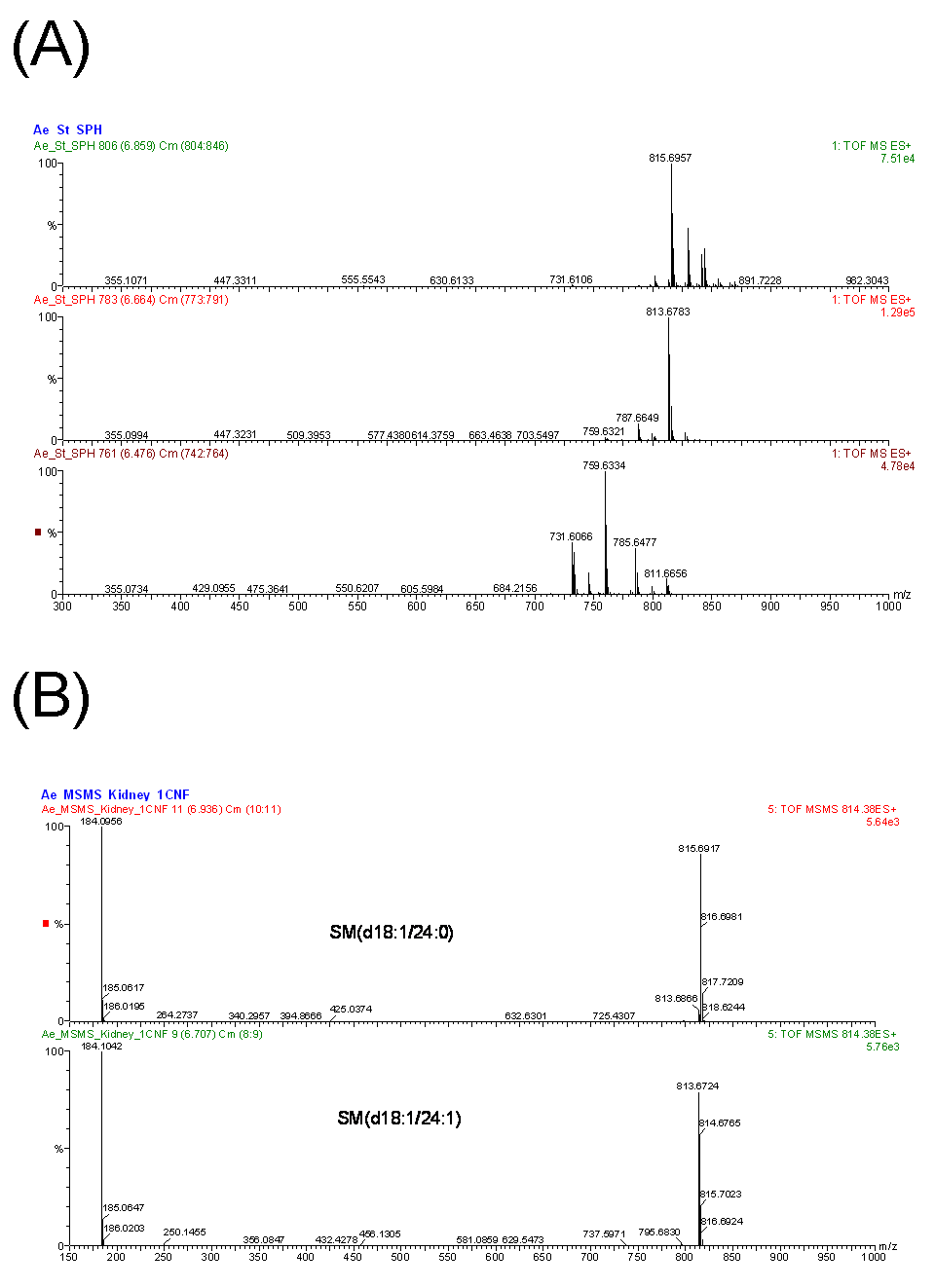

Supplement: Figure S23 — (A) A mixture of ESI+ MS spectra from a sphingomyelin extract. (B) ESI+ MS/MS spectra of two sphingomyelin species. (0.11 MB TIF) [file pone.0000097.s038.tif]
